# Der Antisemitismus der Anderen: Für eine differenzierte Betrachtung antisemitischer Einstellungen unter Muslim:innen in Deutschland

**DOI:** 10.1007/s41682-021-00078-w

**Published:** 2021-09-29

**Authors:** Cemal Öztürk, Gert Pickel

**Affiliations:** 1 University of Duisburg-Essen, Duisburg, Deutschland; 2 University of Leipzig, Leipzig, Deutschland

**Keywords:** Antisemitismus, Vorurteile, Religiöser Fundamentalismus, Rechtspopulismus, Anti-Semitism, Prejudices, Religious Fundamentalism, Right-wing populism

## Abstract

Bereits seit einigen Jahren schwelt eine Diskussion über einen neuen Antisemitismus. Im Fokus dieser Debatten finden sich immer häufiger Einwanderer:innen, vor allem aber Muslim:innen wieder. Als Folge kam der Begriff eines islamisierten Antisemitismus auf. Schnell wurden diese Diskussionen zu einem Politikum. Rechtsextreme Akteure wie die Alternative für Deutschland griffen die Hinweise auf Antisemitismus unter Muslim:innen auf und instrumentalisierten diese für ihre antimuslimische Agenda. Diese Instrumentalisierung wiederum macht es Menschen, die sich gegen antimuslimische Diskriminierung einsetzen, schwer, die Existenz eines muslimischen Antisemitismus anzuerkennen. Anhand unterschiedlichen empirischen Materials untersucht dieser Beitrag die Prävalenz antisemitischer Ressentiments unter Muslim:innen und wie diese mit der Persistenz von Antisemitismus in der deutschen Gesellschaft zusammenhängen. Die Ergebnisse zeigen, dass traditionelle Formen des Antisemitismus und insbesondere israelbezogener Antisemitismus unter Muslim:innen besonders akzentuiert ausfällt. Der Antisemitismus, in muslimischen Submilieus, stellt neben dem ethnonationalen, rechtsextremen Antisemitismus eine Bedrohung für Jud:innen in Deutschland dar. Der Antisemitismus unter Muslim:innen stützt sich sowohl auf Narrative, die aus ihren Herkunftsländern stammen, sowie auf religiöse Quellen. Allerdings ist der Antisemitismus unter Muslim:innen in Deutschland geringer ausgeprägt als in den meisten Gesellschaften der islamischen Welt. Darüber hinaus sind schuldverleugnende Artikulationen von Antisemitismus nach wie vor ein Markenzeichen der autochthonen Bevölkerung und rechter politischer Milieus. Antisemitismus in Deutschland bedarf daher eines differenzierteren Verständnisses, als es noch vor wenigen Jahren notwendig erschien.

## Abstract (en)

For some years now, there has been a discussion about a new anti-Semitism. The focus of these debates is increasingly on immigrants, but above all on Muslims, and thus there is now talk about an Islamized anti-Semitism. These discussions quickly became a political issue. Right-wing extremist actors such as the Alternative for Germany seized on the evidence of anti-Semitism among Muslims and instrumentalized it for their anti-Muslim agenda. This instrumentalization, in turn, makes it difficult for people working against anti-Muslim discrimination to acknowledge the existence of anti-Semitism among Muslims. Using a variety of empirical material, this article examines the prevalence of anti-Semitic resentment among Muslims and how it relates to the persistence of anti-Semitism in German society. The results show that traditional forms of anti-Semitism, and in particular Israel-related anti-Semitism, are particularly accentuated among Muslims. Together with ethnonational, right-wing extremist anti-Semitism, anti-Semitism among Muslim submilieus poses a threat to Jews in Germany. Anti-Semitism among Muslims draws on narratives from their countries of origin and on religious sources. However, anti-Semitism among Muslims in Germany is less pronounced than in most societies in the Islamic world. Moreover, guilt-denying articulations of anti-Semitism remain a hallmark of the autochthonous population and right-wing political milieus. Anti-Semitism in Germany therefore requires a more nuanced understanding than seemed necessary just a few years ago.

## Einleitung: Antisemitismus unter Muslim:innen – Antisemitismus der Gegenwart?

In der langen Historie der Feindschaft gegenüber Jüd:innen spielten religiösen Quellen immer eine beachtliche Rolle. Ausgehend vom christlichen Antijudaismus, der bis in die Neuzeit das Verhältnis der Christ:innen zu den Jüd:innen prägte, entwickelte sich im 19. Jahrhundert ein rassistischer Antisemitismus, der sich im Nationalsozialismus zum industriellen organisierten Völkermord steigerte (u. a. Benz 2016, S. 17–41, 100–141; Bergmann 2016, S. 9–16, 101–115; Brumlik 2020, S. 12–38, 46–70).^1^ In der postnationalsozialistischen Gesellschaft reduzierte sich der offen zur Schau getragener Antisemitismus. Antisemit:innen lernten, wie man Fragen nach Antisemitismus ausweichen konnte. Mit seiner voranschreitenden öffentlichen Unsichtbarkeit, verschwand der Antisemitismus nicht, allenfalls kann von einer Kommunikationslatenz des Antisemitismus die Rede sein (Bergmann und Erb 1986). Kurzum: Man bleibt Antisemit:in, weiß dies aber zu verbergen. Folglich konnten Meinungsumfragen der letzten Jahrzehnte oft nur begrenzte Bestände des tradierten Antisemitismus in der deutschen Gesellschaft identifizieren (u. a. Decker et al. 2012, S. 78, 2018, S. 190–200; Zick et al. 2019, S. 102–110). Für einige der europäischen Nachbarländer sah dies kaum anders aus, selbst bei Berücksichtigung der Besonderheit der jüngeren deutschen Geschichte und des Holocaust (Zick et al. 2011, S. 65–66). Allein die Beobachtung eines sekundären Antisemitismus, der auf Schuldabwehr ausgerichtet ist, säte in Deutschland ein Misstrauen hinsichtlich eines Aussterbens antisemitischer Ressentiments (u. a. Decker et al. 2018; bereits sehr früh Schönbach 1961).^2^

Etwas aus dem Blick geriet dabei ein religiös geprägter Antisemitismus. Dieser erfährt in neuer Form in den letzten Jahren einen Bedeutungszuwachs. Zum einen als Ausdruck eines gegen Israel gerichteten Antisemitismus im arabischen Raum, zum anderen durch Hinweise auf einen Antisemitismus unter Muslim:innen in Europa (Bauer 2018; Brumlik 2020; Jikeli 2017; Ranan 2018; Salzborn 2020). Letzterer wird vor allem als Folge der religiösen Pluralisierung ausgemacht. Mit den Fluchtbewegungen 2015 nahm die Diskussion um einen „importierten Antisemitismus“ zusätzlich Fahrt auf und führte zu Debatten über einen „muslimischen Antisemitismus“ unter Geflüchteten (Arnold und König 2016; Berek 2018).^3^ Allerdings beschränkt sich die Diskussion um einen muslimischen oder islamischen Antisemitismus keineswegs nur auf Geflüchtete, sondern richtet sich im Allgemeinen auf Muslim:innen als Mitglieder einer Religionsgemeinschaft. Neben der Frage, ob es sich dabei um einen durch Herkunft, sozialen Erfahrungen, sozialen Status oder Religiosität geprägten Antisemitismus handle, erfährt die Debatte eine Politisierung. So greifen rechtspopulistische und rechtsextreme Parteien den Verdacht eines unter Muslim:innen grassierenden Antisemitismus auf und instrumentalisieren ihn für ihre Ablehnung von Geflüchteten und Muslim:innen (z. B. Pfahl-Traughber 2017). Auf diese Weise gewinnen sie einen zusätzliches Ansatzpunkt für ihrer Kampagnen gegen Muslim:innen, die ihren Mobilisierungserfolgen europaweit zuträglich sind (Öztürk und Pickel 2019; Pickel und Yendell 2018). Diese Instrumentalisierung erschwert einen offenen Diskurs über die Existenz und Ursachen eines muslimischen Antisemitismus, bzw. einen differenzierten Blick auf die daraus entstehende Zuschreibung (Arnold 2019, S. 147). Nicht selten werden Hinweise auf einen muslimischen Antisemitismus bereits im Vorfeld von Diskussionen als rechte Propaganda geächtet.

Diese Überlegungen führen uns zur Frage: *Findet sich in Deutschland und Europa ein von Muslim:innen ausgehender Antisemitismus im Sinne eines Einstellungsantisemitismus – und in welcher Relation steht dieser zu bereits existierenden Antisemitismen?*

Das Label „muslimisch“ verstehen wir dabei als ein Containerbegriff, der sowohl religiöse, als auch andere Bedeutungselemente beinhalten kann. Er wird in den Bevölkerungsumfragen, die wir empirisch ausgewertet haben, durch die selbst notierte formelle Zugehörigkeit zu einer islamischen Glaubensgemeinschaft bestimmt.^4^ Um einen pauschalisierenden Blick zu überwinden, differenzieren wir, wo es empirisch möglich erscheint, zwischen verschiedenen islamischen Glaubensgemeinschaften. Unsere forschungsleitenden Thesen lauten: *(1) In der Bundesrepublik existieren heute verschiedene Erscheinungsformen des Antisemitismus. (2) Unter Muslim:innen besteht im gesellschaftlichen Vergleich eine überdurchschnittliche Verbreitung antisemitischer Ressentiments, was auch mit Narrativen aus Herkunftsgesellschaften in der islamischen Welt zusammenhängt. (3) Gleichzeitig hat der Antisemitismus religiöse Ursachen: Dogmatisch-fundamentalistische Gläubige, egal ob Christ:innen oder Muslim:innen, neigen zu einer stärkeren Empfänglichkeit für antisemitische Einstellungsmuster. (4) Gerade unter jungen Muslim:innen sind antisemitischer Ressentiments auch ein Produkt ihrer Sozialisation in Deutschland – es kommt zu einer gewissen Übernahme des Antisemitismus der Dominanzgesellschaft. Dieser Anpassungsprozess gilt gleichzeitig in Bezug auf eine geringere Ausprägung des Antisemitismus als in den Herkunftsgebieten eingewanderter Muslim:innen und ihrer Nachkommen.*

Für eine in die Tiefe gehende empirische Behandlung dieses politisch sensiblen Themas beziehen wir unterschiedliche Datenquellen aus der Umfrageforschung in die Analyse ein: Wir verwenden eine Umfrage der *Anti-Defamation League* (2019), den *Konid-2019-Datensatz* (Pickel et al. 2020) sowie Daten der *Leipziger Autoritarismus Studie* (Decker und Brähler 2020). Begleitend beziehen wir uns auf eine Umfrage der *European Union Agency for Fundamental Rights *(FRA 2018) und den *Bertelsmann Religionsmonitor* (Pickel 2020).

## Zeitgenössische Erscheinungsformen des Antisemitismus

### Antisemitismus – eine Phänomenbeschreibung

Folgt man der Definition der International Holocaust Remembrance Alliance (2016), dann ist Antisemitismus eine feindselige Wahrnehmung von Jüd:innen, die in Hass und Gewalt umschlagen können. Antisemitismus richtet sich, in Form von Einstellungen, Worten und Taten gegen jüdisch wahrgenommene Personen, deren Eigentum sowie gegen religiöse Einrichtungen und Institutionen jüdischer Gemeinden. Dabei kann auch der Staat Israel – der von Antisemit:innen als Verkörperung des jüdischen Kollektivs wahrgenommen wird – ins Visier solcher Angriffe geraten. Schäuble (2017, S. 546) bezeichnet den Antisemitismus als Grenzziehung gegenüber einer Konstruktion von „dem Juden“, dessen Konsequenz von einer Gruppenkonstruktion über Stigmatisierung bis zu Gewalt reichen kann. Eine große Rolle spielen dabei Mythen und Zuschreibungsprozesse „of turning Jews into Jews“ (Klug 2003, S. 137).

Somit ist eine zentrale Erscheinungsform des Antisemitismus angesprochen: Der *tradierte Antisemitismus*. Unter ihn fallen alle Einstellungen, die Hinweise darauf liefern, dass Menschen jüdischen Glaubens ein Übermaß an Macht, Zersetzungskraft und internem Zusammenhalt angedichtet werden (Brumlik 2012, S. 66; Kiess et al. 2020, S. 219–220). Als eine der wirkungs-mächtigsten Quellen eines solchen antisemitischen Verschwörungsdenkens gelten die ‚Protokolle der Weisen von Zion‘ – ein Falsifikat, nach dem eine jüdische Geheimgesellschaft nach Weltherrschaft strebt. Jüd:innen werden durch dieses Verschwörungsnarrativ als imaginiertes Kollektiv stigmatisiert und als Drahtzieher allen Übels ausgemacht (Benz 2016, S. 67–68). Antisemit:innen machen sie auf diese Weise zu den Drahtziehern von Dingen, die aus ihrer Sicht nicht richtig laufen (Benz 2016, S. 14). Solche Verschwörungsmythen sind alles andere als ein Relikt der Vergangenheit. Explizit antisemitische Narrative finden sich z. B. in der Vorstellung einer in Washington tonangebenden ‚Israel-Lobby‘ (Beyer 2017). Ein weiteres illustratives Beispiel ist der von Victor Orbán popularisierte Mythos, nach dem George Soros (ein US-amerikanischer Finanzinvestor mit ungarischen Wurzeln und jüdischen Glaubens) in Zusammenarbeit mit Bürokrat:innen der Europäischen Union, die Migrationsströme von 2015 orchestriert habe, um die ‚Islamisierung Europas‘ voranzutreiben (Öztürk und Pickel 2019). Besonders in Krisenzeiten erleben antisemitische Verschwörungslegenden ihre Hochkonjunktur, so kommen die seit der COVID-19-Pandemie florierenden Verschwörungsnarrative selten ohne antisemitische Erzählfiguren aus (Schließler et al. 2020, S. 298–304). Faktisch genießt antisemitisches Verschwörungsdenken also bis heute eine große Popularität. Seine Dauerhaftigkeit und der Umstand, dass Antisemitismus nicht auf realen Erfahrungen aufbaut, sind Gründe, weshalb antisemitische Haltungen als Ressentiment und nicht als basales Vorurteil eingeordnet werden (Kiess et al. 2020, S. 214–215).

Nun wäre es naiv zu glauben, dass Antisemit:innen in Bevölkerungsumfragen eine allzu große Offenbarungsbereitschaft an den Tag legen (Kiess et al. 2020, S. 220). Der Grund hierfür ist simpel: Offen jüd:innenfeindliche Aussagen werden in der Bundesrepublik sanktioniert und rufen zivilgesellschaftliche Gegenwehr hervor. Entsprechend vermeiden viele Antisemit:innen ein offenes Bekenntnis ihres Antisemitismus. Dieser Umstand wird als Kommunikationslatenz bezeichnet (Bergmann und Erb 1986) und sollte nicht darüber hinwegtäuschen, dass in Deutschland ein „Antisemitismus ohne Antisemiten“ (Marin 1979) fortlebt. Antisemit:innen verzichten auf die offene Artikulation ihrer Ansichten, im privaten Raum bestehen antisemitische Weltsichten jedoch fort. Sie treten immer dann in Erscheinung, wenn sich Antisemit:innen alternative Ausdrucksmöglichkeiten bzw. rhetorische Umwege für ihre Ressentiments bieten. Zu dieser Umwegkommunikation gehören der sogenannte sekundäre Antisemitismus sowie der israelbezogene Antisemitismus (u. a. Beyer 2015; Schönbach 1961).

Beim *sekundären Antisemitismus* handelt es sich um eine Sammelbezeichnung für schuld-abwehrende Artikulationen des Antisemitismus (Schönbach 1961). Er ist ein Spezifikum der post-nationalsozialistischen Bundesrepublik und entspringt der Weigerung in die Einsicht, dass sich die eigenen Eltern, Großeltern bzw. Urgroßeltern im Regelfall als aktive Täter:innen oder passive Mitläufer:innen an den Verbrechen des Nationalsozialismus schuldig gemacht haben (Adorno 1997a, b; Salzborn 2020). Der Zorn über die verdrängte Familienerbschaft der Schuld wird auf die Gruppe der Opfer projiziert – es kommt zu einer Täter-Opfer-Umkehrung (Beyer 2015, S. 583–584; Löwenthal 2017, S. 80–82). Sie tritt in Erscheinung, wenn Jüd:innen eine Mitschuld an der Shoah oder Instrumentalisierung des Holocaust angedichtet wird und die Verbrechen des Nationalsozialismus durch Relativierungen und Relationssetzungen bagatellisiert werden. Zum Schuldabwehrantisemitismus gehört eine Überdramatisierung des eigenen Leid – schließlich waren die Bombardierungen deutscher Städte und die Vertreibung von Deutschen eine Reaktion auf den Vernichtungskrieg der Nationalsozialisten (Rensmann 2004, S. 162–170; Kiess et al. 2020, S. 220).

Der *israelbezogene Antisemitismus* ist ein weiterer rhetorischer Umweg, der es Antisemit:innen erlaubt, ihre antijüdischen Ressentiments offen zu kommunizieren. Er ist weitestgehend salonfähig, weil die Feindschaft gegenüber Jüd:innen im Gewand einer ‚Kritik‘ am Staat Israel daher kommt (Beyer 2015, S. 584–585; Brumlik 2020, S. 71–77). Damit ist nicht gesagt, dass eine Kritik an der israelischen Regierung illegitim ist. Um eine sachliche Kritik geht es beim israelbezogenen Antisemitismus jedoch nicht. Vielmehr werden im Fahrwasser vermeintlicher Israel-Kritik antisemitische Ressentiments und Stereotype kommuniziert, die mit der zu kritisierenden Angelegenheit nichts mehr zu tun haben (Benz 2016, S. 190). Letzteres ist zu beobachten, wenn die Kritik an der israelischen Regierung als Feigenblatt für kollektive Ressentiments gegen Jüd:innen genutzt werden (Klug 2003). Dies ist der Fall, wenn Gleichsetzungen mit dem nationalsozialistischen Deutschland vorgenommen werden, das Existenzrecht Israels generell bestritten oder mit doppelten Standards hinsichtlich bestimmter ‚Verfehlungen‘ im Ländervergleich argumentiert wird. Gut sichtbar wird der als Israel-Kritik getarnte Antisemitismus an den drei Indikatoren der Dämonisierung, Delegitimierung und Doppelstandards (Sharansky 2013; Salzborn 2020). Gelegentlich wird die vermeintliche Israel-Kritik mit schuldabwehrenden Artikulationen des Antisemitismus verquickt. Wenig überraschend finden sich dann auch Belege, dass es sich bei israelbezogenen Antisemitismus um eine empirische Dimension des Antisemitismus handelt (Kiess et al. 2020, S. 231). Der Grund für eine zunehmende Verbreitung des israelbezogenen Antisemitismus liegt in seiner panideologischen Anschlussfähigkeit: Der als Israel-Kritik getarnte Antisemitismus hat eine Tradition in antiimperialistischen Bewegungen und linken Submilieus (Benz 2016; Beyer 2017; Haury 2019; Poliakov 1992; Rickenbacher 2020). Er ist darüber hinaus aufgrund seiner diskursiven Verbindung mit schuldabwehrenden Artikulationen des Antisemitismus unter Wähler:innen des rechten Parteienspektrums weit verbreitet (Salzborn 2018; Rensmann 2020). Gleich mehrere Studien verzeichnen zudem akzentuierte Zustimmungswerte zum israelbezogenen Antisemitismus unter Muslim:innen (Decker und Celik 2019; Pickel et al. 2020) – womit sich nun die Frage nach den Besonderheiten des Antisemitismus unter Muslim:innen stellt.

### Geschichte und Charakteristiken des muslimischen Antisemitismus

Gleich zu Beginn ist zu konstatieren, dass es keinen anerkannten Begriff zur Kennzeichnung des von muslimischen Submilieus ausgehenden Antisemitismus gibt (Kiefer 2006). Gängig ist der Versuch eine klare Grenze zwischen dem Islam und dem Islamismus zu ziehen. Antisemitismus wird dann als Folge des Islamismus ausgewiesen. Diese Grenzziehung folgt der Intention, die in Deutschland lebenden Muslim:innen vor weiteren Stigmatisierungen zu schützen (Becker 2020, S. 76), denen sie aufgrund von antimuslimischen Rassismus und Muslim:innenfeindlichkeit unterworfen sind (statt vieler Pickel und Yendell 2016, Shooman 2014; Zick 2017). Gänzlich überzeugend, ist diese Grenzziehung allerdings nicht.

Der Antijudaismus unter Muslim:innen besitzt eine lange Tradition und es existiert eine beachtliche Verbreitung des Antisemitismus in der islamischen Welt. Dies wäre ohne europäischen Einfluss und transnationalen Transfer gar nicht möglich gewesen (Becker 2020, S. 78–81; Kiefer 2006; Tibi 2017). Wenig umstritten ist allerdings auch, dass es für den Antijudaismus unter Muslim:innen im Koran zumindest Anhaltspunkte gibt (Kaddor et al. 2019, S. 20). Für ein besseres Verständnis der Genealogie dieses islamisierten Antisemitismus ist es sinnvoll zwischen dem traditionellen Antijudaismus vormoderner islamischer Gesellschaften und dem modernen Antisemitismus des 20. Jahrhundert zu differenzieren (Küntzel 2020).

Traditionell ist das Verhältnis des Islams zum Judentum ist von einer gewissen Ambivalenz geprägt. Auf der einen Seite hat der Islam viele prophetische Traditionen des Juden- und Christentums in seine heiligen Schriften integriert. Muslim:innen sind ferner dazu aufgerufen Jüd:innen sowie Christ:innen als ‚Völker des Buches‘ zu tolerieren (Becker 2020, S. 78; Kaddor et al. 2019; Tibi 2017). Auf der anderen Seite verdankte sich der weltlich-machtpolitische Aufstieg des Propheten Mohammed auch eines militärischen Sieges über in Medina ansässige jüdische Stämme. Eine Konsequenz dieser Auseinandersetzungen ist, dass sich im Koran einige Stellen finden, in denen Gewalt gegen Jüd:innen legitimiert wird. Letztere werden z. B. in Sure 5:60 als Affen und Schweine enthumanisiert (Kaddor et al. 2019, S. 20). In Sure 9:29 wird zu einem Kampf gegen die ‚Völker des Buches‘ aufgerufen, der solange geführt werden soll, bis sie Tribut zollen und sich unterwerfen (Salzborn 2018, S. 120). Auch sind die zuvor erwähnten Aufrufe zur Toleranz gegenüber Jüd:innen keinesfalls mit einem egalitären Status zu verwechseln. Jüd:innen wurde ein sogenannter Dhimmi-Status gewährt. Sie galten als Schutzbefohlene, sie mussten Sondersteuern bezahlen und sie waren als Menschen zweiter Klasse gesellschaftlicher Ächtung ausgesetzt (Bostom 2008; Küntzel 2020; Jikeli 2015; Tibi 2017).

Nun muss an dieser Stelle betont werden, dass dem traditionelle Antijudaismus in der islamischen Welt zu keiner Zeit dieselbe Intensität wie dem christlichen Antijudaismus innewohnte. Der Dhimmi-Status bot Jüd:innen in islamisch begründeten Herrschaftssystemen einen gewissen Schutz. Im Vergleich zum christlichen Europa waren Jüd:innen seltener Pogromen ausgesetzt. So wurde beispielsweise al-Andalus – der muslimisch beherrschte Teil der iberischen Halbinsel – zu einem Refugium für Jüd:innen. Nach der ‚Reconquista‘ flüchtete der Großteil der sephardischen Jüd.innen in das osmanische Reich und in den Maghreb (Brumlik 2018; Lewis 1984). Die Vorstellung eines goldenen Zeitalters islamischer Toleranz wäre trotzdem eine unzulässige Verklärung (Becker 2020). Auch in islamischen Herrschaftsgebieten entlud sich der Antijudaismus in tödliche Gewaltexzesse (Kaddor et al. 2019, S. 34; Jikeli 2019, S. 61). Selbst die formale Abschaffung des Dhimmi-Status im 19. Jahrhundert änderte wenig für sie. Jüd:innen waren in ihrem alltäglichen Leben in vielen arabischen Gesellschaften systematischer Gewalt ausgesetzt (Bensoussan 2019; Fenton und Littman 2016).

Der traditionale Antijudaismus in den islamischen Gesellschaften hatte allerdings gegenüber dem christlichen Europa eine Besonderheit. Da die Truppen des Propheten Mohammeds über die jüdischen Stämme in Medina siegten, galten ‚die Juden‘ als inferior, während sie im christlichen Europa – auch weil sie für den Tod von Jesus verantwortlich gemacht wurden – geradezu als ‚kosmisches Übel‘ eingestuft wurden (Becker 2020, S. 76; Küntzel 2020). Durch das Aufkeimen des modernen Antisemitismus im 19. zum 20. Jahrhundert erfuhren die gängigen Erzählungen über ‚die Juden‘ in der islamischen Welt einen Wandel. Eine bedeutende Rolle spielte die Übersetzung von antisemitischen Hetzschriften in die Sprachen des Vorderen Orients (Küntzel 2020). Aus den als ‚unterlegen‘ angesehenen Juden wurden ‚omnipotente Juden‘, die das Ziel verfolgen, dem Islam und seinen Anhänger:innen zu schaden (Becker 2020, S. 76).

Zu einer flächendenkenden Verbreitung solcher antisemitischen Verschwörungsmythen trug ab den 1930er-Jahren die Propaganda der Nationalsozialisten bei (Herf 2009). Nazi-Deutschland verfolgte im arabischen Raum zwei Ziele. Zum einem erhofften sich die Nationalsozialist:innen in der arabischen Welt einen geschlossenen Widerstand gegen die Gründung Israels zu mobilisieren. Zum anderen eröffnete Adolf Hitler dem Führer der palästinensischen Nationalbewegung Amin el-Husseini, die Aussicht die Shoah auf die 700.000 Jüd:innen des arabischen Raumes auszuweiten (Küntzel 2020; Mallmann und Cüppers 2010). Um für diese Zielsetzung Unterstützer:innen zu finden, bemühte sich Nazi-Deutschland den Antisemitismus in der islamischen Welt anschlussfähig zu machen.^5^ Dieser deutsche Export blieb nicht ohne Folgen, wie ein Blick auf die in den 1920er-Jahren gegründete Muslimbruderschaft zeigt. Nach dem Ende des Zweiten Weltkrieges avancierte sie – auch dank einer finanziellen und ideologischen Unterstützung durch die Nationalsozialisten – zu einer der einflussreichsten Massenbewegungen der arabischen Welt. Keine sechs Monate nach dem Ende des Zweiten Weltkrieges organisierte die Muslimbruderschaft anti-jüdische Pogrome in Kairo und Alexandria. Ferner trug sie durch ihre Massenmobilisierung dazu bei, dass die ägyptische Armee im Zusammenschluss mit Transjordanien, Syrien, dem Irak und dem Libanon dem Staat Israel am Tag seiner Gründung den Krieg erklärte. Dass gleich fünf arabische Armeen einen militärischen Sieg Israels nicht vermeiden konnten, war für viele Araber:innen eine nur schwer zu ertragende Schmach und führte zu einer gesteigerten Empfänglichkeit für antisemitische Verschwörungsmythen (Küntzel 2020).

Eine simple Deutung für die desolate Lage lieferte der damalige Chefideologe der Muslimbruderschaft: *Sayyid Qutb*. Sein Traktat ‚Unser Kampf mit den Juden‘ gilt als ein Schlüsseltext des Islamismus und hat maßgeblich zu einer Popularisierung des islamisierten Antisemitismus beigetragen (Tibi 2017, S. 122–129). Qutb lieferte eine klare Feindbildkonstruktion. Schuld an der Misere seien ‚die Juden‘. Sie hätten sich seit Mohammeds Ankunft in Medina gegen den Islam verschworen und würden sich erst mit seiner Zerstörung zufriedengeben. Qutb konstruiert die Idee einer ewigen Feindschaft zwischen Islam und Judentum, der zugleich ein kosmischer Kampf zwischen Gut und Böse ist – wobei ‚die Juden‘ das Böse schlechthin verkörpern. Unterschiede zwischen der israelischen Regierung, der israelischen Gesellschaft, dem Zionismus und dem Judentum werden nicht gemacht (Becker 2020, S. 80–81; Küntzel 2020; Jikeli 2019, S. 65; Tibi 2017, S. 129). Nachdem im Zuge des Sechs-Tagekrieges (1967) ein weiterer Versuch Israel von der Landkarte zu tilgen scheiterte, verbreitete das saudische Königshaus die Schriften Qutbs in der islamischen Welt (Bauer 2018, S. 26). Seine Schrift hat auch deshalb das Denken islamistischer Denker:innen – und zwar weit über die Kernländer der islamischen Welt hinaus – stark geprägt (Jikeli 2015, S. 204–205). Der von ihm popularisierte ‚islamisierte Antisemitismus‘ gehört bis heute zur Kernideologie der Muslimbruderschaft. Al-Qaradawi vertritt die Auffassung, dass ein Dialog mit Jüd:innen nur mit ‚Schwertern‘ und ‚Gewehren‘ möglich ist (Tibi 2017, S. 122). Im TV-Sender Al-Jazeera bezeichnete er Adolf Hitler als einen Gesandten Allahs (Küntzel 2020). Mehr als das: Die Ideen von Qutb sind praxisrelevant geworden, so finden sich z. B. in der Charta der jihadistischen Hamas viele Bezüge zu seinem antisemitischen Traktat (Wyss 2020, S. 73–75).

Der moderne Antisemitismus ist auch in der islamischen Welt panideologisch anschlussfähig und hat – so muss betont werden – auch in den vergleichsweisen *säkularen Ideologien* des panarabischen Nationalismus seinen Anklang gefunden. Hervorzuheben ist die Rolle von *Gamal Abdel Nasser*. Nach seiner Machtübernahme gewährte Ägypten einigen Nazi-Kriegsverbrechern Unterschlupf. Louis Heiden und Johann von Leers übersetzten die ‚Protokolle der Weisen von Zion‘ und ‚Mein Kampf‘ ins Arabische. Leopold Gleim – ein ehemaliger Gestapoführer in Polen – übernahm einen hohen Posten im ägyptischen Sicherheitsapparat. Über Abdel Nasser ist dokumentiert, dass er den Holocaust leugnete und seinen Mitstreiter:innen die ‚Protokolle der Weisen von Zion‘ zur Lektüre empfahl. Ähnliche Sympathiebekundungen gegenüber dem Nationalsozialismus und antisemitische Wahnvorstellungen sind über die syrische und irakische Bath-Partei dokumentiert. Erwähnenswert ist auch der gestürzte lybische Machthaber Gaddafi. Seit seiner Machtübernahme 1973 unterstützte Gaddafi kriegerische Akte gegen den Staat Israel und terroristische Attentate auf jüdische Einrichtungen weltweit (Jikeli 2015, S. 200–201; siehe auch Tibi 1997; Litvak und Webman 2009). Den französischen Holocaustleugner Roger Garaudy zeichnete der lybische Machthaber mit seinem „Gaddafi-Preis für Menschenrechte“ aus (Croitoru 2011).

Ein Extrembeispiel außerhalb der arabischen Welt ist die iranische Republik. Seit der islamischen Revolution von 1979 gehören Antisemitismus und die aktive Bekämpfung Israels – etwa durch die finanzielle und logistische Unterstützung der Hisbollah – zur iranischen Staatsräson (Jikeli 2015, S. 205). Erwähnenswert ist in diesem Zusammenhang auch die vom ehemaligen Präsidenten Ahmadinijad organisierte Holocaustleugnungs-Konferenz (Litvak 2006; Michael 2007).

Auch die Geschichte der Türkei ist nicht frei von Antisemitismus. Die antijüdischen Pogrome der 1930er-Jahren und eine Reichtumssteuer für Jüd:innen (sowie Christ:innen) in den 1940er-Jahren gehören – neben dem Völkermord an den Armenier:innen – zu den dunkelsten Kapitel der Türkei (Anderson 2009, S. 55, 114, auch Pekesen 2014). Antisemitismus ist bis heute weitverbreitet und gehört zu den Kernideologien von extrem-rechten und islamistischen Gruppierungen (Jikeli 2015, S. 191, auch Aviv 2017; Bali 2013). Zu den ideologischen Vordenkern des türkischen Rechtsextremismus gehören u. a. Ziya Gökalp, Nihal Atsiz, und Alparslan Türkes – dem Gründer der Nationalistischen Bewegungspartei (MHP), dessen paramilitärische Arm, die Grauen Wölfe, als eine der größten rechtsextremen Organisationen in Deutschland bezeichnet werden kann. In der Ideologie des türkischen Rechtsextremismus – der letztlich auf einer Synthese von Türkentum und sunnitischem Islam beruht – nimmt der Antisemitismus eine dezidiert rassistische Färbung an, weil den Türk:innen eine Überlegenheit gegenüber anderen Ethnien zugesprochen wird (Bozay 2017; Bozay und Mangitay 2016, S. 13, 25–27). Unter türkischen Islamist:innen ist es gängig den Untergang des osmanischen Reiches und die Errichtung der modernen Türkei unter Kemal Atatürk als einen ‚jüdischen Komplott‘ darzustellen (Jikeli 2015, S. 191).

Hervorzuheben ist in diesem Kontext die von Necmettin Erbakan gegründete – und auch in Deutschland aktive – Islamische Gemeinschaft Mili Görüs (IGMG). Ihre Ideologie der ‚gerechten Ordnung‘ ist dezidiert antifeministisch, homophob, antisemitisch und antiliberal. Demokratie wird als Mittel der Machterlangung akzeptiert, der ihr innewohnende Pluralismus jedoch abgelehnt (Schmidinger 2020). Zu einer Popularisierung des Antisemitismus hat auch der amtierende Präsident Erdogan – der als politischer Ziehsohn Erbakans bezeichnet werden kann – beigetragen. Seine Sympathie für den Dichter Necip Fazıl Kısakürek ist bestens dokumentiert (Singer 2013). Aus der Feder von Kısakürek stammen politische Pamphlete, in denen Jüd:innen, aber auch andere ethnische und religiöse Minderheiten als hinterlistige Feinde der Türkei ausgemacht werden (Jikeli 2015, S. 192). Passend hierzu macht Erdogan eine ominöse ‚Zinslobby‘ für die Gezi-Proteste, aber auch den Verfall der türkischen Lira verantwortlich und bedient sich somit eindeutig antisemischer Chiffren (Baer 2017). Antisemitismus ist zudem fester Bestandteil alltäglicher Popkultur. Serien wie ‚Payitaht-Abdul Hamid‘, die antisemitische Stereotype verbreiten, erfreuen sich in der Türkei großer Beliebtheit und werden von Vertreter:innen der türkischen Regierungspartei öffentlich angepriesen (Tokatli und Yilmaz 2019).

### Forschungsleitende Annahmen zum muslimischen Antisemitismus

Antisemitismus ist in der islamischen Welt also alles andere als eine Randerscheinung und weist über die Ränder islamistischer Milieus hinaus. Antisemitismus ist Bestandteil von Alltagsdiskursen und wird in regierungsnahen wie in oppositionellen Kreisen, im Fernsehen, als auch in Predigten perpetuiert (Becker 2020, S. 81). Aus sozialisationstheoretischer Perspektive ist es äußerst unwahrscheinlich, dass dies keine Konsequenzen für das geistige Klima in den jeweiligen Gesellschaften hat. *Wir gehen deshalb davon aus, dass antisemitische Einstellungsmuster in islamischen Gesellschaften im globalen Vergleich deutlich prävalenter ausfallen als in Deutschland (These 4). *Gleichzeitig liegt die Vermutung nahe, dass auch unter den deutschen Muslim:innen (noch) stärke Zustimmungswerte zu antisemitischen Aussagen verzeichnet werden können.* Es liegt die Vermutung nahe, dass antisemitischen Einstellungsmuster unter Muslim:innen in Deutschland höher ausfallen als im Mainstream der deutschen Dominanzgesellschaft (These 2)*. Hinweise für diese Annahme liefern kulturvergleichende Wertestudien von Norris und Inglehart (2012) sowie von Alexander und Welzel (2011). Sie können am Beispiel von Werten, die Sexualmoral und Geschlechterbeziehungen betreffen, aufzeigen, dass sich Muslim:innen mit einer Zuwanderungsgeschichte – meist ab der zweiten Generation – von dem Wertegravitationszentrum ihrer Herkunftsländer distanzieren und dem Gesellschaftsklima ihrer neuen Heimat anpassen. Ihre Ergebnisse zeigen jedoch auch, dass signifikante Unterschiede zum Mainstream der Dominanzgesellschaft bestehen bleiben.

Die *intergenerationale Transmission* von antisemitischen Narrativen in Familien dürften hierbei Konsistenzen schaffen. Allerdings ist in Rechnung zu stellen, dass Muslim:innen, die zum Teil bereits in der vierten Generation in Deutschland leben, in einer Gesellschaft sozialisiert werden, die den Antisemitismus – entgegen verschiedener Selbstdarstellungen – noch nicht überwunden hat (Arnold 2020; Decker et al. 2018; Kiess et al. 2020; Messerschmidt 2021; Zick et al. 2019; Zick und Küpper 2021). Die Wahrnehmung eines tradierten oder sekundären Antisemitismus in der deutschen Bevölkerung harmoniert dabei mit erlernten Vorstellungen von Jüd:innen. *So gehen wir davon aus, dass antisemitische Ressentiments gerade unter jungen Muslim:innen auch ein Produkt ihrer Sozialisation in Deutschland sind und dass es zu einer gewissen Anpassung an den Antisemitismus der Dominanzgesellschaft kommt (These 4). *Der Einfluss antisemitischer Alltagsdiskurse aus der ‚alten Heimat‘ sollte dabei nicht unterschätzt werden. Auch in Deutschland war die islamistische Zeitung Vakit, die Necmettin Erbakans Mili Görüs-Bewegung nahestand, lange Zeit am Kiosk erhältlich. Sie wurde 2005 aufgrund antisemitischer Hetzpropaganda in Manier des Stürmers verboten (Jikeli 2015, S. 2017).^6^ Im Zeitalter der Globalisierung und transnationaler Mediennutzung (siehe Unabhängiger Expertenkreis Antisemitismus 2017, S. 79) kann der Internetauftritt einer solchen Zeitung jedoch nicht unterbunden werden. Gleiches gilt u. a. für Serien wie ‚Payitaht-Abdul Hamid‘, schließlich wird der türkische Staatssender TRT auch in deutschen Wohnzimmern empfangen.

Hinzu kommt der Faktor der ‚*Religion*‘ oder der Einfluss großer Islamverbände. Dass in einer Hamburger Mili Görüs-Moschee (IGMG) in der Vergangenheit ein Hetzvideo auftauchte, das offenen Judenhass propagierte (Hamburg Behörde für Inneres und Sport 2006), vermag vor dem Hintergrund ihres Spiritus Rectors Necmettin Erbakan – der bis heute verehrt wird – kaum überraschen. Auch andere deutsche Islamverbände stehen in der Tradition von Organisationen, in denen der Antisemitismus die Rolle einer Kernideologie einnimmt. So gehört die Deutsche Muslimische Gemeinschaft (DMG) zum Netzwerk der Muslimbruderschaft (Breuer 2019). Die Union der Türkisch-Islamischen Kulturvereine in Europa e. V. (ATIB) gilt als islamistischer Flügel der rechtsextremen Grauen Wölfe (Bozay und Mangitay 2016, S. 59–62). Problematisch ist darüber hinaus die Einflussnahme von autokratischen Regimen. Die Islamische Gemeinschaft der schiitischen Gemeinden Deutschlands (IGS) und das Islamische Zentrum Hamburg (IZH) sind dem obersten Geistlichen der Islamischen Republik Iran unterstellt (Bundesministerium des Innern, für Bau und Heimat 2019, S. 201). Die Türkisch-Islamische Union der Anstalt für Religion (DITIB) wird vom türkischen Ministerium für religiöse Angelegenheiten kontrolliert – weshalb ein Bedeutungsgewinn islamistischer Ideologie keinesfalls ausgeschlossen werden kann (Jikeli 2015, S. 208). Nun wäre es falsch den genannten Organisationen eine allzu große Wirkung auf die deutschen Muslim:innen zu attestieren, schließlich fühlt sich nur ein Viertel der in Deutschland lebenden Muslim:innen von den Islamverbänden adäquat repräsentiert (Bundesamt für Migration und Flüchtlinge 2009, S. 179). Gleichwohl ist davon auszugehen, dass *antisemitische Einstellungsmuster unter Muslim:innen vor allem dann akzentuierter ausfallen, wenn sie zu einer dogmatisch-fundamentalistischen Auslegung ihrer Religion tendieren (These 3). *Als Folge dieser Betrachtungen müssten* sich antisemitische Ressentiments in Deutschland in einer größeren Vielfalt in unterschiedlichen Gruppen manifestieren (These 1).*

## Ausgangsbeobachtungen: Betroffenenperspektiven und die Verbreitung antisemitischer Ressentiments im internationalen Vergleich

### Die Perspektive der Betroffenen: Antisemitische Diskriminierungserfahrungen und Bedrohungswahrnehmungen von Jüd:innen in Deutschland

Um sich dem zu untersuchenden Phänomen anzunähern, lohnt sich ein kleiner Umweg. In den meisten Debatten zum Antisemitismus in den letzten Jahrzehnten wurde Menschen jüdischen Glaubens zumeist eine passive Rolle zugewiesen, es wurde in aller Regel über sie gesprochen und sie selbst kamen selten zu Wort (siehe auch Chernivsky et al. 2020). Erst in jüngerer Zeit gibt es den Versuch mehr über die Situation und die Einschätzungen von Jüd:innen zu erfahren. Dies erlaubt nicht nur eine Sensibilisierung für die Gefahrenlage, der Menschen jüdischen Glaubens in Deutschland bis heute ausgesetzt sind, sondern auch einen Blick auf Antisemit:innen aus der Betroffenenperspektive (Beyer und Liebe 2020, S. 137–141).

**Figure 1:**
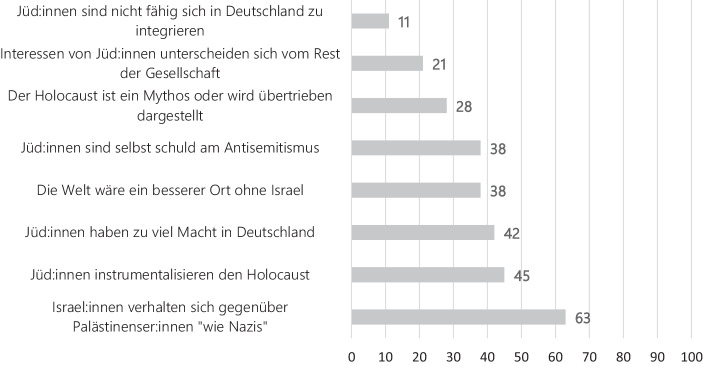

So gibt eine Umfrage der *European Union Agency for Fundamental Rights *(FRA 2018) über Diskriminierungserfahrungen und antisemitische Hasskriminalität, an der sich 1233 Menschen jüdischen Glaubens beteiligt haben, einen alarmierenden Einblick über antisemitische Aussagen mit denen Jüd:innen in ihrem Alltag konfrontiert werden (Abb. 1). Ihnen wird zwar selten kommuniziert, dass sie nicht in die deutsche Gesellschaft integrierbar seien, doch haben ca. 20 % der Befragten schon einmal zu hören bekommen, dass sie doch ‚anders‘ sind und sich stark vom Rest der Gesellschaft unterscheiden. Dies zählt noch zu harmlosesten Aussagen: 38 % wurden schon mal mit der Aussage konfrontiert, dass sie selbst die Verantwortung für den Antisemitismus tragen. 42 % haben persönlich erlebt, dass ihnen zu viel ‚Macht über Deutschland‘ nachgesagt wird. 45 % der Befragten wurde schon vorgehalten, dass ‚die Juden‘ den Holocaust instrumentalisieren – und dass letzterer doch eventuell ein Mythos sei oder zumindest übertrieben dargestellt werde. Ganz besonderer Beliebtheit erfreut sich zudem eine Konfrontation mit dem Nahostkonflikt. 63 % der Befragten wurde schon einmal entgegnet, dass das Verhalten von Israel:innen gegenüber den Palästinenser:innen ‚dem Vorgehen‘ der Nazis ähnele. Es kommt also zu einer *Schuldumkehr*. Deutlich wird: *Jüd:innen sind in ihrem alltäglichen Leben immer wieder mit Täter-Opfer-Umkehrungen, klassischen antisemitischen Stereotypen, NS-Verharmlosungen, Holocaustrelativierungen und israelbezogenem Antisemitismus konfrontiert*.

**Figure 2:**
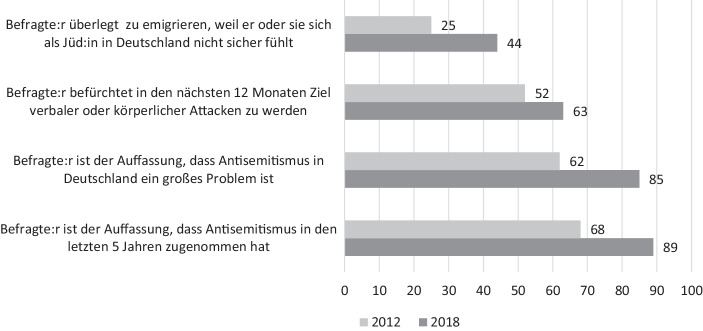

Diese Erfahrungen gehen nicht spurlos an ihnen vorbei. Mehr als 80 % der europäischen Jüd:innen haben den Eindruck, dass der Antisemitismus in den letzten Jahren an Intensität gewonnen hat. Die Tendenz ist steigend, wenn man Befragungen aus den Jahren 2012 und 2018 miteinander ins Verhältnis setzt – was dann in Teilen auch die Debatten über einen ‚neuen Antisemitismus‘ erklärt (Heilbronn et al. 2019; Lipstadt 2019). 60 % der Befragten halten es für realistisch in den nächsten 12 Monaten zum Ziel verbaler oder körperlichen Attacken zu werden. Und mehr als das: 44 % der Befragten haben schon mal einen Gedanken darauf verwendet aus Deutschland auszuwandern, weil sie sich in diesem Land nicht mehr sicher fühlen (Abb. 2). Nimmt man diese Aussagen zusammen, dann wird deutlich: *Zwischen dem häufig kolportierten Selbstbild einer deutschen Gesellschaft, die den Antisemitismus überwunden hat, und den Erfahrungen die Jüd:innen in ihrem Leben machen, klafft eine beachtliche Lücke.*

**Figure 3:**
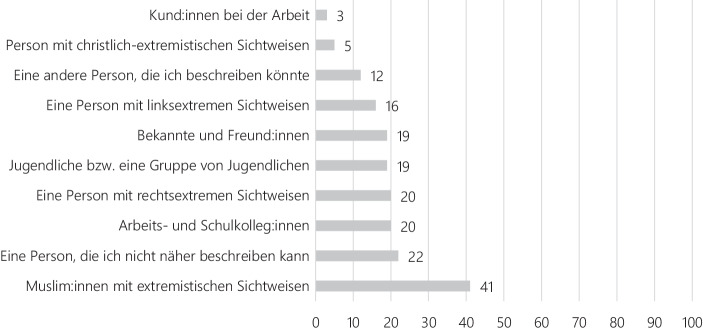

Doch von welchen Personen gehen solche antisemitischen Belästigungen nun aus? Auch für diese Frage finden sich in der Umfrage der European Union Agency for Fundamental Rights (FRA 2018) Anhaltspunkte. Befragte, die direkte persönliche Diskriminierungserfahrungen gemacht haben – dies waren zum Zeitpunkt des Surveys 28 % der Befragten – wurden nach einer Beschreibung des Täter:innenprofils gebeten (Abb. 3). Ein Blick auf die Antworten zeigt, dass Antisemitismus ein gesamtgesellschaftliches Problem darstellt.

Nicht einmal der persönliche Nahbereich ist vor antisemitischen Vorfällen gefeit. 19 % der Befragten berichten, dass sie von Bekannten und sogar Freund:innen mit antisemitischen Stereotypen und Vorurteilen konfrontiert wurden. Eine fast ebenso große Anzahl der Befragten nennt Arbeits- und Schulkolleg:innen als Ausgangspunkt antisemitischer Anfeindungen. Zu einem Alltagsantisemitismus im Nahbereich passt auch, dass sich Gruppen von Jugendlichen unter den Nennungen befinden. Relativ häufig werden Personen mit linksextremistischen (16 %) Sichtweisen und rechtsextremistischen Weltbildern (20 %) genannt.

Aus der Breite der genannten Profile wird deutlich: *Antisemitismus ist in Deutschland panideologisch anschlussfähig* (Beyer und Liebe 2020, S. 141; Rensmann 2020; Salzborn 2018). Diese Aussage bezieht auch religiöse Gruppen mit ein. Vergleichsweise selten werden Täter:innen genannt, die aus einer christlich-extremistischen Motivation (5 %) heraus handeln. Ein christlicher Antijudaismus ist zwar nicht verschwunden, aber doch eher ein Randphänomen. *Die Gruppe, die von den Betroffenen jedoch mit großem Abstand am häufigsten genannt wurde, sind Muslim:innen mit extremistischen Auslegungen ihrer Religion (41* *%).*

Betroffenenbefragungen unterliegen gewissen Verzerrungen: Menschen werden aufgrund askriptiver Merkmale als muslimische Täter:innen beschrieben. Ein direkter Rückschluss von ihren askriptiven Merkmalen auf ihre ideologische Motivlage ist nicht eins zu eins möglich (Zick et al. 2017, S. 21). Zudem weist die Erfassung antisemitischer Hasskriminalität durch die amtliche Polizeistatistik in eine andere Richtung: 90 % aller registrierten antisemitischen Vorfälle gehen auf das Konto von Rechtsextremist:innen (Bundesministerium des Innern, für Bau und Heimat 2018).

Diese Hinweise unterliegen allerdings der Gefahr den von Muslim:innen ausgehenden Antisemitismus zu unterschätzen. Zwei Gründe sind hierfür zu nennen: Zum einem existiert eine große Dunkelziffer von antisemitischen Vorfällen, die von den Betroffenen nicht zur Anzeige gebracht werden (FRA 2018, S. 45). Zum anderen sind die erwähnten Polizeistatistiken in die Kritik geraten und mit etwas Vorsicht zu verwenden. Die ideologischen Hintergründe antisemitischer Straftaten in Deutschland werden nicht systematisch erfasst – Straftaten aus muslimischen Milieus nicht gesondert ausgewiesen. Unaufgeklärte Fälle werden dem Phänomenbereich ‚politisch motivierter Kriminalität‘ zugeordnet (Steinke 2020, S. 95, sie auch Jüdische Allgemeine 2019).

Der Survey der European Union Agency for Fundamental Rights (FRA 2018) ist nicht die einzige Betroffenenbefragung. Ein vom Institut für interdisziplinäre Konflikt- und Gewaltforschung (IKG) durchgeführter Online-Survey, an dem 533 Menschen jüdischen Glaubens teilgenommen haben, kommt zu ähnlichen Ergebnissen. *Auch hier finden sich Muslim:innen unter den am häufigsten genannten Personengruppen, von denen verdeckte antisemitische Andeutungen, verbale Beleidigungen und körperliche Angriffe ausgehen. Aus Sicht der Betroffenen lag der Anteil von Täter:innen mit islamischer Religionszugehörigkeit bei körperlichen Angriffen sogar bei 81* *%* (Zick et al. 2017, S. 21). Grund genug um zu vermuten, dass jüdisches Leben im Deutschland des 21. Jahrhundert, neben dem Rechtsextremismus und dem Alltagsantisemitismus, auch durch den islamisierten Antisemitismus bedroht wird.

Nun wäre es fatal die in Deutschland lebende Muslim:innen als Kollektiv für diese gewalttätigen Erscheinungsformen des Antisemitismus verantwortlich zu machen. Auslegungen des Islams sind schließlich genauso vielfältig, wie die in Deutschland lebenden Muslim:innen. Gleichwohl existieren scheinbar Gruppen unter ihnen, die antisemitischen Ressentiments stärker zuneigen. Antisemitische Hasskriminalität – egal welcher ideologischen Provenienz – entsteht zudem nicht in einem gesellschaftlichen Vakuum. Offensichtlich finden sich in unter den in Deutschland lebenden Muslim:innen einige Submilieus, die antisemitische Orientierungsmuster tolerieren bzw. aktiv kultivieren (Jikeli 2019, S. 51). Als Gründe für den von Muslim:innen ausgehenden Antisemitismus wird in aller Regel sowohl auf religiöse als auch auf migrationsbezogene Quellen verwiesen (Decker und Celik 2019, S. 59). Letzterer Verweis zielt auf die (ehemaligen) Herkunftsländer der in Deutschland lebenden Muslim:innen und die Annahme, dass der Antisemitismus dann teilweise weitersozialisiert wird. Wie steht es aber um die Verbreitung des Antisemitismus in der islamischen Welt?

### Sind antisemitische Einstellungsmuster in islamischen Gesellschaften weiter verbreitet? Ein internationaler Vergleich auf Grundlage einer Befragung der Anti-Defamation League

Damit setzen wir an unserer ersten forschungsleitenden Hypothese an. Zu ihrer Überprüfung nutzen wir wie bereits Tausch (2014) eine Umfrage der Anti-Defamation League (2019).^7^ Im Rahmen des Surveys wurden elf gängige antisemitische Stereotype abgefragt. Die Fragen umfassen im Regelfall Aussagen, die dem tradierten Antisemitismus zugeordnet werden können, da sie darauf schließen lassen, dass die Befragten dazu tendieren, Jud:innen eine omnipotente Machtposition zu zusprechen. Zu diesen Fragen zählen Aussagen wie „Juden sind für die meisten Kriege in der Welt verantwortlich“ oder „Die Juden kontrollieren die globalen Medien“. Hinzu kommt der Vorwurf, dass „Juden loyaler gegenüber Israel als gegenüber ihren Heimatländern sind“ oder die Klage, dass „Juden zu häufig den Holocaust thematisieren“. Ferner umfasst der Survey Items, die Jüd:innen abschätzige Charaktereigenschaften attestieren: Beispielhaft sei die Aussage „Juden denken sie seien besser als anderen Menschen“ genannt. Abschließend wird die Empfänglichkeit für Täter-Opfer-Umkehrungen mit der Aussage „Juden sind an dem Hass der ihnen entgegenschlägt selber schuld“ erfasst. Basierend auf diesen Fragen wird ein Antisemitismus-Index erstellt, der Auskunft über den Anteil der Bevölkerung gibt, die mindestens sechs der elf Stereotype für ‚vermutlich wahr‘ halten (Anti-Defamation League 2019).

**Figure 4:**
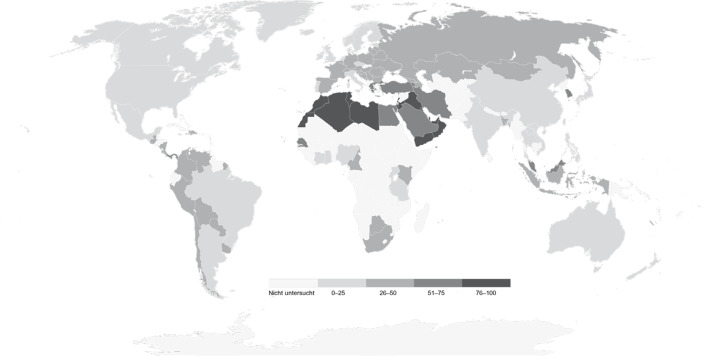

Einen ersten Hinweis auf die stärkere Prävalenz antisemitischer Einstellungen in der islamischen Welt liefert die Heatmap in Abb. 4 (siehe auch Tausch 2014). Das empirische Muster fällt eindeutig aus: In der Gruppe der Nationen, in denen weniger als 50 % der Bevölkerung sechs der elf antisemitischen Stereotypen zustimmen, sind Gesellschaften mit mehrheitlich muslimischer Bevölkerung mit sieben von insgesamt 78 Fällen stark unterrepräsentiert. Zu diesen Staaten gehören Nigeria, die Elfenbeinküste, Bangladesch, Bosnien-Herzegowina, Kasachstan, Aserbaidschan und Indonesien – und somit Gesellschaften die nicht zu den islamischen Kernländern gezählt werden können. Vice versa gilt, dass in Staaten, in denen mindestens jede:r zweite:r Befragte:r den antisemitischen Stereotypen zustimmt, Gesellschaften mit einer mehrheitlich islamischen Bevölkerung mit 20 von 24 Fällen überrepräsentiert sind. In der Gruppe der Staaten, in denen mindestens 2 von 3 Befragten – also über 68 % der Bevölkerung – der Mehrheit der antisemitischen Items zustimmt, ist Griechenland die einzige nicht-islamische Gesellschaft. Besonders prävalent ist das antisemitische Gesellschaftsklima in der Türkei und arabischen Gesellschaften. In den Vereinigten Arabischen Emiraten, Katar, Marokko, Jordanien, Bahrain, Kuwait, Tunesien, Libyen, Algerien und im Jemen stimmen mehr als 80 % der Bevölkerung einer Mehrheit der Items zu. Im Irak und Palästina übersteigen die Zustimmungswerte sogar die 90-%-Marke.

Wie sind diese Ergebnisse zu deuten? Ist ein prävalentes antisemitisches Gesellschaftsklima eventuell gar keine Besonderheit islamischer Gesellschaften, sondern von arabischen Gesellschaften? Steckt hinter den akzentuierten antisemitischen Ressentiments – wie David Ranan (2018) argumentiert – vor allem der ungelöste Territorialkonflikt zwischen Palästina und Israel? Tatsächlich sind vor allem die arabischen Gesellschaften Hochburgen eines antisemitischen Gesellschaftsklima. Es wäre also ziemlich abenteuerlich dem Nahostkonflikt jegliche Bedeutung abzusprechen. Aus unserer Sicht darf die Rolle dieses territorialen Konfliktes jedoch auch nicht überschätzt werden. Einseitige Verweise auf das Konfliktgeschehen im Nahen Osten tragen dazu bei, antisemitische Ressentiments grundsätzlich zu rationalisieren. Jüd:innen wird durch diese Argumentation eine Mitschuld an der Entstehung von Antisemitismus gegeben – und zwar völlig unabhängig von der Frage, ob sie in Israel leben oder nicht. Im schlimmsten Fall führt diese Argumentation zu einer Täter-Opfer-Umkehrung (Jikeli 2015, S. 199).

**Figure 5:**
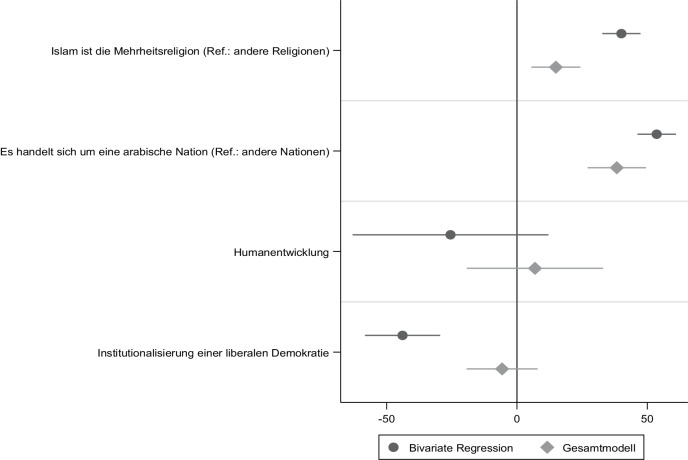

Auch empirisch spricht vieles dafür, dass der Nexus zwischen einer mehrheitlich muslimischen Bevölkerung und einem antisemitischen Gesellschaftsklima keine Besonderheit arabischer Nationen darstellt. In Abb. 5 finden sich die Ergebnisse einer OLS-Regression, in der für die Erklärung eines antisemitischen Gesellschaftsklimas – bemessen am Antisemitismus-Index der Anti-Defamation League (2019) – neben einer mehrheitlich muslimischen Bevölkerung weitere Faktoren aufgenommen wurden. Selbst wenn wir kontrollieren, ob die untersuchten Gesellschaften in der arabischen Welt (β = 39,84, *p* = 0,0001) liegen, bleibt der signifikante Effekt einer mehrheitlich muslimischen Bevölkerung auf ein antisemitisches Gesellschaftsklima (β = 13,90, *p* = 0,001) bestehen (siehe Tausch 2014).

Dieser Zusammenhang ist robust und bleibt auch unter Kontrolle der gesellschaftlichen Humanentwicklung (β = 4,53, *p* = 0,730) und der im Sample variierenden institutionellen Verwirklichung der liberalen Demokratie (β = −4,35, *p* = 0,535) bestehen. Keine Bestätigung findet somit die modernisierungstheoretische Annahme, dass gesellschaftlicher Wohlstand automatisch zu einer größeren Toleranz gegenüber Minoritäten führt (z. B. Lipset 1959). Auch die Etablierung einer liberalen Demokratie – deren Gesetze und Verfassung zur Ächtung diskriminierender Positionen gegenüber Minderheiten beitragen sollten (Merkel 2004) – wirken antisemitischen Ressentiments nicht signifikant entgegen. Bivariat besteht allerdings ein solcher Effekt (β = −25,11, *p* = 0,0001), im Gesamtmodell wiegt die gesellschaftlichen Mehrheitsreligion jedoch schwerer.

Weniger technisch ausgedrückt: Auch in den demokratischen Staaten der islamischen Welt (z. B. dem Senegal oder Indonesien) sind die Bürger:innen für antisemitische Stereotype empfänglicher als in nicht-islamischen Gesellschaften mit ähnlichem Demokratisierungs- und Humanentwicklungsniveau. Somit kann die erste forschungsleitende These nicht verworfen werden. Ein antisemitisches Gesellschaftsklima ist in den meisten islamischen Gesellschaften nicht die Ausnahme, sondern der Regelfall (siehe auch Beyer 2019; Jikeli 2019; Solomon und Tausch 2020; Tausch 2014). Allerdings handelt es sich hierbei um Aussagen über das *sozial-dominante geistige Klima* in den untersuchten Gesellschaften. Rückschlüsse auf Individuen und Muslim:innen in europäischen Einwanderungsgesellschaften sind auf Grundlage aggregierter Zustimmungswerte nicht möglich. Gleichwohl liefern die Daten der Anti-Defamation League (2019) erste Hinweise auf die Plausibilität unserer zweiten These – also der Annahme, dass antisemitische Einstellungsmuster auch in Einwanderungsgesellschaften wie Deutschland (und anderen europäischen Nationen) unter Muslim:innen verbreiteter sind als im Mainstream der Dominanzgesellschaft.

**Figure 6:**
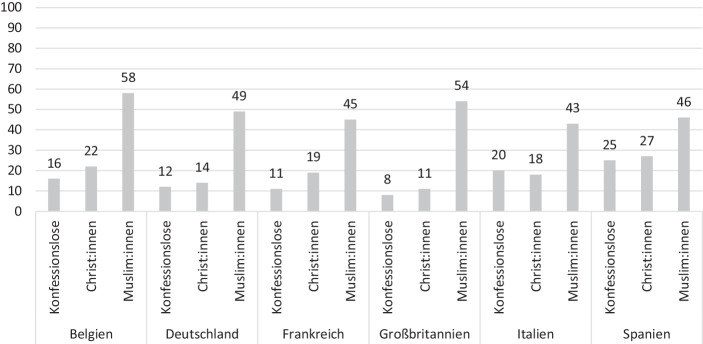

2019 wurde in Belgien, Deutschland, Frankreich, Großbritannien, Italien, und Spanien, im Zuge eines weiteren Surveys, zusätzlich ein Sample von 100 Muslim:innen interviewt (siehe Anti-Defamation League 2019). Zwar lässt das Sample von 100 Befragten keine repräsentativen Aussagen über ‚die Muslim:innen‘ in Europa zu. Allerdings verfestigen die Ergebnisse der Anti-Defamation League (2019) ein empirisches Muster, welches auch andere Studien zeigen: Antisemitische Ressentiments sind unter Muslim:innen verbreiteter als unter Nicht-Muslim:innen (u. a. Brettfeld und Wetzels 2007; Koopmans 2015). Wie der Abb. 6 entnommen werden kann, halten die interviewten Muslim:innen in allen sechs untersuchten Gesellschaften die antisemitischen Stereotype öfter für vermutlich wahr. Das Niveau der Empfänglichkeit für antisemitische Ressentiments ist im Durchschnitt zwei Mal höher als unter Christ:innen oder Konfessionslosen – wobei die Zustimmungswerte unter den Muslim:innen in Europa gleichzeitig deutlich niedriger ausfallen als in der Türkei oder in arabischen Gesellschaften. Offensichtlich sind ‚cross-pressures‘ am Werk: Auf der einen Seite gelingt eine gewisse Transmission erlernter antisemitischer Narrative aus der Herkunftsgesellschaften. Auf der anderen Seite kommt es zu einem Abbau offen geäußerter antisemitischer Ressentiments, da in den europäischen Untersuchungsstaaten Antisemitismus geächtet wird. Gleichzeitig werden – um an dieser Stelle einen Befund vorweg zu nehmen – auch in den europäischen Einwanderungsgesellschaften fortexistierende antisemitische Narrative aufgegriffen.

Es lassen sich drei empirische Muster festhalten: (1) Antisemitismus ist in der großen Mehrheit islamischer Gesellschaften ein sozial-dominantes Einstellungsmuster und im globalen Vergleich besonders stark verbreitet. (2) Muslim:innen tendieren auch in Einwanderungsgesellschaften häufiger zu antisemitischeren Einstellungsmustern als der Mainstream der Gesellschaft. (3) Dabei passen sich Muslim:innen über die Zeit dem Wertegravitationszentrum ihrer gesellschaftlichen Umgebung an – es bleiben jedoch signifikante Unterschiede im Vergleich zum Mainstream der Gesellschaft bestehen.

## Tiefenanalysen in Deutschland – Muslimischer Antisemitismus als „der neue Antisemitismus“?

### Die Verteilung antisemitischer Ressentiments in Deutschland

Nun verlangen die präsentierten Befunde nach einer Kontextualisierung. Es stellt sich die Frage in welcher Relation der Antisemitismus unter Muslim:innen und der fortexistierende Antisemitismus in der Dominanzgesellschaft zu einander stehen? Ein sensibles und facettenreiches Erhebungsinstrument für antisemitische Einstellungsmuster liefert die Datenerhebung der *Leipziger Autoritarismus-Studie* (Decker et al. 2020, S. 27–40). Dies liegt zum einem an der Erhebungsmethode und zum anderen an den Items mit denen antisemitische Orientierungsmuster erhoben werden. Bei der Umfrage der Leipziger Autoritarismus-Studie, die zwischen Mai und Juni 2020 vom Meinungsforschungsinstitut USUMA erhoben wurden, kam das sogenannte Paper-Pencil-Vorgehen zum Einsatz. Die Datengenerierung basiert somit nicht auf einer mündlichen Befragung oder Telefoninterviews. Die Befragten haben stattdessen den inhaltlichen Teil des Fragebogens eigenständig beantwortet, ohne dass die Interviewenden – die für Rückfragen zur Verfügung stehen – Kenntnis von den Antworten erhalten. Dieses Vorgehen begünstigt eine höhere Offenbarungsbereitschaft (Decker et al. 2020).

Hinzukommt, dass die Leipziger Autoritarismus-Studie (Decker und Brähler 2020) ein detailliertes Erhebungsinstrument zur Erfassung antisemitischer Orientierungsmuster liefert. Es umfasst insgesamt 12 Items mit denen drei Dimensionen des Antisemitismus abgebildet werden können. Hierbei handelt es sich um den tradierten Antisemitismus, den tradierten Antisemitismus in der Umwegkommunikation, den Schuldabwehrantisemitismus und den israelbezogene Antisemitismus (Tab. 1). Die Items des tradierten Antisemitismus in der Umwegkommunikation bieten den Befragten dabei eine Möglichkeit ihre (tradierten) Ressentiments gegen Jüd:innen indirekt zu äußern. Eine weitere Besonderheit der Erhebung liegt in dem Umstand, dass den Befragten die Antwortkategorie „stimme teils zu, teils nicht zu“ zur Verfügung steht. Diese Antwortkategorie liefert den Befragten die Möglichkeit ihre Unentschiedenheit zu kommunizieren. Diese Nichtablehnung von klaren antisemitischen Statements wird von den Durchführenden der Studie als Hinweis auf das latente Potenzial des Antisemitismus verwendet und gibt Rückschlüsse auf seine Verbreitung in Deutschland (Kiess et al. 2020, S. 224).^8^

**Table 1:** 

Dimensionen	Items	Manifeste Zustimmung (%)	Latente Zustimmung (%)
*Tradierter* *Antisemitismus*	Auch heute noch ist der Einfluss der Juden zu groß	11,1	24,2
	Die Juden arbeiten mehr als andere Menschen mit üblen Tricks	7,5	19,6
	Die Juden haben einfach etwas Besonderes und Eigentümliches passen nicht so recht zu uns	6,2	18,4
*Tradierter* *Antisemitismus (Umweg-kommunikation)*	Ich kann es gut verstehen, dass manchen Leuten Juden unangenehm sind	11,3	23,4
	Über die Juden sollte man besser nicht sprechen	8,9	20,5
	Juden gehören selbstverständlich zur deutschen Bevölkerung	11,3	22,7
*Schuldabwehrantisemitismus*	Es macht mich wütend, dass die Vertreibung der Deutschen und die Bombardierung deutscher Städte immer als kleinere Verbrechen angesehen werden	33,5	34,6
	Reparationsforderungen an Deutschland nützen oft gar nicht den Opfern, sondern einer Holocaust-Industrie von findigen Anwälten	40,2	31,9
	Wir sollten uns lieber gegenwärtigen Problemen widmen als Ereignissen, die mehr als 70 Jahre vergangen sind	55,5	23,6
*Israelbezogener Antisemitismus*	Israels Politik in Palästina ist genauso schlimm wie die Politik der Nazis im Zweiten Weltkrieg	29,6	38,4
	Durch die israelische Politik werden mir die Juden immer unsympathischer	13,2	29,1
	Auch andere Nationen mögen ihre Schattenseiten haben, aber die Verbrechen Israels wiegen am schwersten	11,6	33,5

*Quelle*: Leipziger Autoritarismus-Studie (Decker und Brähler 2020). Anmerkung: Die Antwortkategorien ‚stimme überwiegend zu‘ und ‚stimme voll und ganz zu‘ werden als manifeste Zustimmung gewertet. Die Antwortkategorie ‚stimme teils zu, teils nicht zu‘ wird als latente Zustimmung gewertet. Eigene Darstellung.

**Figure 7:**
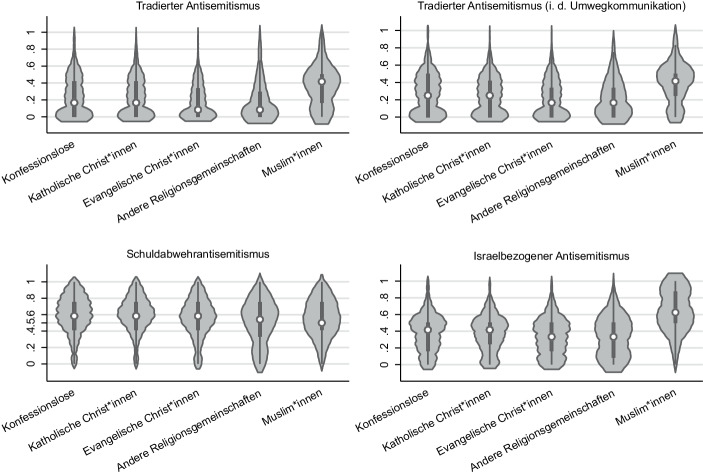

Tab. 1 gibt einen Überblick über die 12 verwendeten Items und ihre manifest und latenten Zustimmungswerte in Deutschland. Der manifeste tradierte Antisemitismus schwankt zwischen 6 und 11 %. In aller Regel wählen mindestens zwei bzw. drei von zehn Befragten die Antwortkategorie „stimme teils zu, teils nicht zu“. Deutet man das entlang der bisherigen Argumentation, so liefert dies Hinweise auf eine nicht zu unterschätzende *Kommunikationslatenz des Antisemitismus* (Bergmann und Erb 1986). Gleichzeitig ist der tradierte Antisemitismus bei weitem nicht mehr die zentrale Form antisemitischer Ressentiments. Die modale Erscheinungsform des Antisemitismus ist in Deutschland der Schuldabwehrantisemitismus. Die manifesten Zustimmungswerte fallen bei dieser Facette des Antisemitismus mit Abstand am höchsten aus. Da die Leipziger Autoritarismus-Studie (Decker und Brähler 2020) auch eine Frage zur Konfession der Befragten umfasst, sind vergleichende Betrachtungen zwischen Muslim:innen und Nicht-Muslim:innen möglich. Wie den Violin-Plots in Abb. 7 entnommen werden kann, fällt die Zustimmung zum tradierten Antisemitismus unter Muslim:innen im Vergleich zu Mitgliedern anderen Religionsgemeinschaften und Konfessionslosen akzentuierter aus.

Zu einer gewissen Nivellierung dieser Unterschiede kommt es, wenn der tradierte Antisemitismus in der *Umwegkommunikation* in den Blick genommen wird. Die Mittelwertdifferenzen zwischen Muslim:innen und Mitgliedern anderen Religionsgemeinschaften sowie Konfessionslosen fallen jedoch auch bei dieser Facette des Antisemitismus signifikant aus.

Da wir für die Mittelwertvergleiche die Skalen auf einen Bereich von 0 bis 1 normalisiert haben und sowohl der Median als auch der Mittelwert in der Gruppe der Muslim:innen deutlich unter dem Wert 0,50 liegt – also der Bereich der Skala, der als mindestens latente Zustimmung gewertet werden kann – muss auch hier betont werden, dass die Empfänglichkeit für den tradierten Antisemitismus in der Gruppe der Muslim:innen keinesfalls mehrheitsfähig ist. Anders verhält es beim israelbezogenen Antisemitismus. Bei dieser Facette des Antisemitismus treten die Unterschiede zwischen Muslim:innen und vergleichbaren Gruppen der Dominanzgesellschaft am deutlichsten zu Tage. Nicht nur die Mittelwertdifferenzen zwischen den kontrastierend untersuchten Gruppen fallen signifikant aus. Es ist sogar die Mehrheit der Befragten Muslim:innen, die mindestens latent und in großen Teilen sogar manifest zu einer Dämonisierung, Delegitimierung und doppelten Standards gegenüber Israel tendiert.

Die skizzierten empirischen Muster werden jedoch brüchig, wenn die modale Erscheinungsform des Antisemitismus in den Blick genommen wird. Der Schuldabwehrantisemitismus ist ein Charakteristikum der Dominanzgesellschaft. Bemerkenswert ist, dass die Haltungen der Muslim:innen zum Schuldabwehrantisemitismus – trotz der deutlichen Mittelwertunterschiede – nicht signifikant von der Dominanzgesellschaft abweichen. Ob es sich hierbei um die Konsequenz einer Sozialisation in Deutschland oder um eine Anpassung der eigenen antisemitischen Ressentiments an den deutschen Kontext handelt, kann an dieser Stelle nicht endgültig beantwortet werden. So oder so: Der Befund bietet Raum für Reflexionen. Erstens widerspricht dieses empirische Muster dem medial kolportierten Bild des ‚importierten Antisemitismus‘ (Gensing und Reisin 2019). Mit diesem Begriff wird suggeriert, dass Antisemitismus in erster Linie eine Konsequenz von Einwanderung sei und quasi mitmigriert. Wenn aber die große Mehrheit der in Deutschland lebenden Muslim:innen den Schuldabwehrantisemitismus der Dominanzgesellschaft internalisiert hat – und darauf deuten die nicht-signifikanten Mittelwertunterschiede hin – dann liegt die Vermutung nahe, dass der Antisemitismus unter Muslim:innen nicht nur ‚fremden Ursprungs‘ ist. Viele der befragten Muslim:innen sind in Deutschland aufgewachsen und wurden in deutschen Kindergärten und Schulen sozialisiert. Der Antisemitismus der vermeintlich Anderen ist somit eben auch ein Versagen der Dominanzgesellschaft und ihrem Umgang mit der Geschichte (Arnold 2020). Deutschland ist gut beraten den Antisemitismus unter Muslim:innen – bei dem es sich ja beim genaueren Hinsehen auch um einen europäischen Export handelte – als den seinigen zu realisieren und Haltungen statt Herkunft in den Fokus der Aufmerksamkeit rücken. Für die realen Bedrohungen von Jüd:innen ist es nämlich am Ende des Tages zweitrangig, ob der in Deutschland existierende Antisemitismus importiert oder hausgemacht ist (Arnold 2019, S. 137).

Damit ist eine weitere Problemlage angesprochen. Es entsteht nämlich zweitens der Eindruck, dass der in den letzten Jahren so intensiv diskutierte ‚Antisemitismus der Anderen‘ (Hagen und Neuburger 2020, S. 9–15) zu einer Projektionsfläche avanciert ist (Rohde 2019).

Bei allen Differenzen im Meinungsklima zwischen Muslim:innen und Nicht-Muslim:innen fällt auf, dass der israelbezogene Antisemitismus und insbesondere der Schuldabwehrantisemitismus – der die Signatur der Dominanzgesellschaft trägt – weit in den Mainstream der deutschen Gesellschaft reicht. Nimmt man diesen gesellschaftlichen Kontext in den Blick, dann stellt die Frage, *ob die große Aufmerksamkeit für den islamisierten Antisemitismus nicht einer Verharmlosung von antisemitischen Ressentiments der Mehrheitsbevölkerung den Weg ebnet*. Der Antisemitismus wird auf eine gesellschaftlich marginalisierte Gruppe externalisiert, was mit dem praktischen Nebeneffekt einhergeht, dass sich Deutschland als eine vom Antisemitismus geläuterte Gesellschaft inszenieren kann (Arnold 2019; Czollek 2018).

Insbesondere die Alternative für Deutschland (AfD) hat sich die strategische Dividende dieses Diskurses in den letzten Jahren zu eigen gemacht. Im Zuge des Bundestagswahlkampfes von 2017 inszenierte sich die AfD immer wieder als Garant für ein sicheres jüdisches Leben in Deutschland und beklagte einen vermeintlich ‚neuen‘ Antisemitismus in Deutschland, der durch die Zuwanderung muslimischer Geflüchteter bedingt sei.

**Figure 8:**
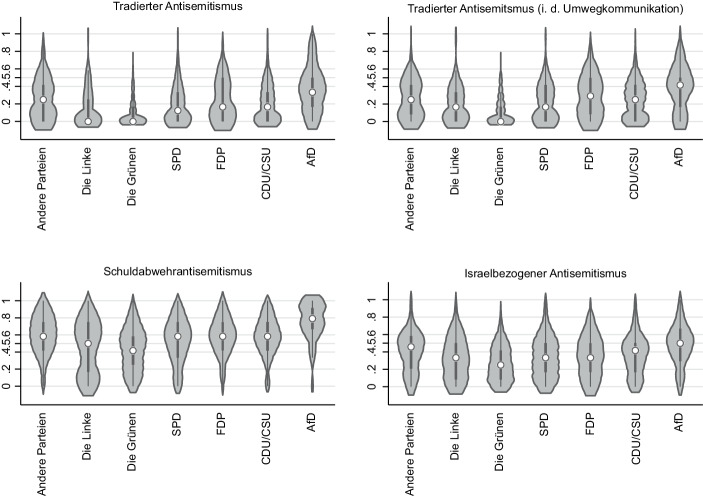

Die Vorteile dieser Rhetorik liegen auf der Hand: Die AfD kann sich mit dem vermeintlichen Anti-Antisemitismus dem Vorwurf des Rechtsextremismus entledigen und ihren muslim:innenfeindlichen Grenzziehungen eine bürgerlich-liberale Patina verleihen (Pfahl-Traughber 2019, S. 19, Rohde 2019, S. 65). Die mangelnde Authentizität dieses Auftretens ist bereits dechiffriert. Ein Grund hierfür sind antisemitische Äußerungen führender AfD-Politiker:innen (Salzborn 2019). Der Fall des – mittlerweile aus der Partei ausgeschlossenen – baden-württembergischen Landtagsabgeordneten Wolfgang Gedeon, der das Falsifikat der ‚Protokolle der Weisen von Zion‘ für echt erklärt und Holocaust-Leugner als ‚Dissidenten‘ bezeichnet, ist dabei nur die Spitze des Eisberges (Pfahl-Traughber 2019, S. 19–20, Rohde 2019, S. 62). Letzterer fußt auf dem Fundament einer Partei, die der Erinnerungs- und Geschichtspolitik der Bundesrepublik den Kampf angesagt hat (Salzborn 2020). Erwähnenswert ist in diesem Kontext Björn Höcke, der das Berliner Denkmal für die ermordeten Jüd:innen als „Mahnmal der Schande“ bezeichnete (Kamann 2017), als auch Alexander Gauland, der es als redlich empfindet auf die „Leistungen deutscher Soldaten in zwei Weltkriegen“ wieder stolz sein zu dürfen (Frankfurter Rundschau 2017). Die mitschwingende Verharmlosung des Nationalsozialismus – schließlich geht es um den Stolz auf Soldaten, die einen (antisemitischen) Vernichtungskrieg geführt haben – ist als manifester Schuldverweigerungsantisemitismus zu bezeichnen. Zwischen dem ostentativ vor sich hergetragenen Anti-Antisemitismus und den Einstellungen der AfD-Wähler:innenschaft klafft zu dem eine gewaltige Lücke (Schuler et al. 2018). Wie Abb. 8 entnommen werden kann, ist das Wähler:innensegment der AfD für alle untersuchten Facetten des Antisemitismus sichtbar empfänglicher als die Wähler:innenschaft der anderen Parteien. Es ließe sich resümieren: Je stärker die Betonung des Antisemitismus der Anderen, desto stärker die Abwehr einer kritischen Selbstreflexion über den Antisemitismus in den eigenen Reihen.

**Figure 9:**
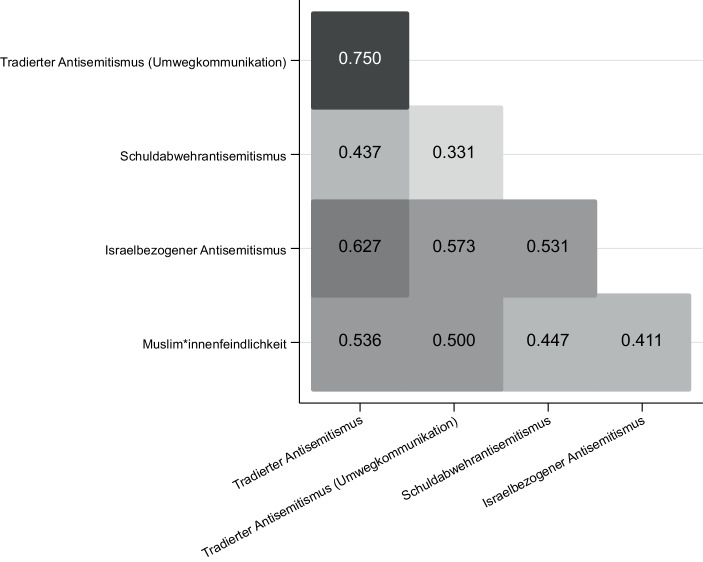

Das inszenierte Bild einer vermeintlich vom Antisemitismus geläuterten deutschen Gesellschaft, die ein Unbehagen gegenüber den ‚antisemitischen Muslim:innen‘ hegt, wird jedoch noch an einer anderen Stelle brüchig. In der Dominanzgesellschaft – und dieses Problem weist weit über die AfD hinaus – liegt eine Verknüpfung von antisemitischen und muslim:innenfeindlichen Ressentiments vor. Auf Basis von vier gegen Muslim:innen gerichtete Aussagen wurde ermittelt, ob die Befragten den Muslim:innen eine größere Neigung zur Kriminalität attestierten; gleiche Rechte absprechen; sich aufgrund ihrer Anwesenheit wie Fremde im ‚eigenen Land‘ fühlen und sich für ein Zuwanderungsverbot für Muslim:innen aussprechen. Die Zustimmung zu diesen Aussagen haben wir zu einer Muslim:innenfeindlichkeits-Skala zusammengesetzt und mit den vier Facetten des Antisemitismus in Verbindung gesetzt. Die Korrelations-Heatmap in Abb. 9 bestätigt einmal mehr die Befunde der Studie zum autoritären Charakter: „wer Feindschaft zeigt gegenüber einer Minderheitengruppe, hegt sie wahrscheinlich auch gegen die meisten anderen“ (Adorno 1976, S. 12).

Empirisch lässt sich beobachten, dass zwischen muslim:innenfeindlichen Ressentiments und gängigen Erscheinungsformen des Antisemitismus, wie dem tradierten Antisemitismus (r = 0,536, *p* = 0,0001), dem tradierten Antisemitismus in der Umwegkommunikation (r = 0,500, *p* = 0,0001), dem Schuldabwehrantisemitismus (r = 0,447, *p* = 0,0001) sowie dem israelbezogenen Antisemitismus (r = 0,411, *p* = 0,0001) eindeutige Bezüge bestehen. Mit diesem Befund harmonieren rechtsextreme und offen antisemitisch konnotierte Verschwörungstheorien, nach der dunkle Mächte die Zuwanderung von Geflüchteten orchestriert haben, um einen ‚jüdischen Umvolkungsplan‘ in die Tat umzusetzen.

Dieser Umstand – und die doch beachtlich verbreitete Muslim:innenfeindschaft in Deutschland und Europa (Strabac und Listhaug 2007; Pickel und Öztürk 2018; Yendell und Pickel 2020) – dürfte zugleich der Grund dafür sein, warum Teile des linken bzw. linksliberalen Milieus der Problematik des islamisierten Antisemitismus mit einer gewissen Scheu begegnen. Da Muslim:innen in Deutschland und Europa selbst mit Rassismus konfrontiert sind, ist man gewillt die Gruppe der Muslim:innen vor weiterer Stigmatisierung zu schützten (Becker 2020, S. 76). Antisemitismus wird allenfalls im äußersten radikalen Rand muslimischer Milieus verortet (Rickenbacher 2020).

Am Ende des Tages ist jedoch weder die Instrumentalisierung noch die Bagatellisierung des islamisierten Antisemitismus ein adäquater Umgang mit dem Antisemitismus. Da Nazi-Deutschland eine treibende Kraft bei der Formation des islamisierten Antisemitismus war (Harf 2009), ist es die historische Pflicht dieses Landes einen – ohnehin schon längst in Gang gesetzten – Reflexionsprozess in muslimischen Communities zu unterstützen und der Verbreitung des Antisemitismus entgegen zu wirken (Becker 2020, S. 84).

### Differenzierungen des Antisemitismus unter Muslim:innen

**Figure 10:**
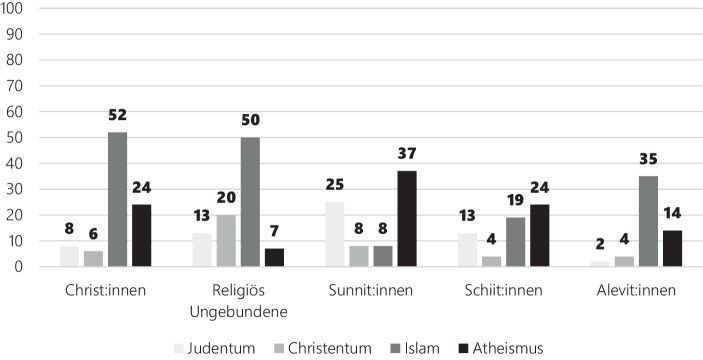

Wie eingangs betont, ist es geboten einen differenzierten Blick auf die deutschen Muslim:innen einzunehmen. Bevölkerungsumfragen liefern dabei in aller Regel nur gröbere Einordnungen. Letzteren können durchschnittliche Unterschiede zwischen den kontrastierten Gruppen entnommen werden. Dies wird nicht der *Heterogenität* der jeweiligen Gruppen gerecht. Zudem bleiben die Gründe für die beachtlichen Unterschiede der Antisemitismusempfänglichkeit *innerhalb* der Gruppen durch Verweise auf Durchschnittswerte und andere Lagemaße der zentralen Tendenz unaufgeklärt. Gleichwohl kann man seine Betrachtungen feiner parzellieren. So liefert dann der Religionsmonitor der Bertelsmann Stiftung Hinweise auf beträchtliche Unterschiede zwischen den Anhänger:innen verschiedener islamischen Glaubensrichtungen (Pickel 2020, S. 84). In einem Survey von 2017 wurden die Bürger:innen befragt, ob sie verschiedene religiöse bzw. nicht religiöse Weltanschauungen als Bedrohung empfinden (Abb. 10). Das stark abweichende Antwortverhalten der verschiedenen Gruppen offenbart das gesellschaftliche Konfliktpotenzial der voranschreitenden religiösen Pluralisierung. Während etwa die Hälfte der Befragten, die sich mit christlichen Konfessionen identifizieren oder angeben religiös ungebunden zu sein, den Islam als Bedrohung empfinden, ist es unter Sunnit:innen und Schiit:innen der Atheismus, der die stärksten Bedrohungsperzeption evoziert. Besonders hervorzuheben sind jedoch die Unterschiede zwischen den Anhänger:innen der verschiedenen islamischen Glaubensrichtungen mit Blick auf die Bedrohungsperzeptionen gegenüber dem Judentum. Während ein Viertel der Sunnit:innen die Existenz des Judentums als bedrohlich empfindet, sind es unter den Schiit:innen 13 % und unter den Alevit:innen sogar nur 2 % der Befragten.^9^

Da die Intoleranz der Alevit:innen gegenüber dem Judentum deutlich geringer ausfällt als unter Christ:innen und religiös ungebundenen Individuen, tragen pauschalisierende Aussagen über ‚die‘ Muslim:innen wenig zu einem Erkenntnisgewinn bei (Pickel 2020, S. 85).^10^

**Figure 11:**
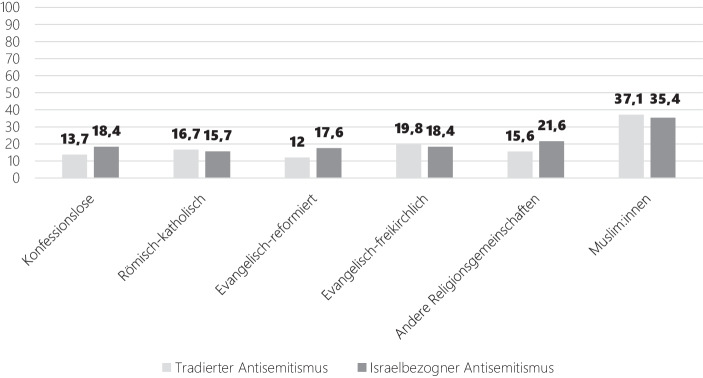

Damit stellt sich auch die Frage, ob die in den vorangegangenen Kapiteln berichteten Unterschiede zwischen Muslim:innen und Nicht-Muslim:innen mit anderen Drittvariablen erklärt werden können. Um diese Frage zu beleuchten, greifen wir auf eine Bevölkerungsumfrage zurück, die im Rahmen eines Projektes zu den Konfigurationen sozialer und religiöser Identitäten (KONID) entstanden ist (siehe Pickel et al. 2020; Liedhegener et al. 2021). Der Survey umfasst neben einer bevölkerungsrepräsentativen Gesamtstichprobe eine Sonderstichprobe über Personen, die aus der Türkei oder dem Iran stammen oder direkte Nachfahren dieser Einwanderergruppe sind (*n* = 580). Da im Rahmen der Studie zwei Fragen gestellt wurden, mit denen sich der tradierte (‚Auch heute noch ist der Einfluss der Juden zu groß‘) und israelbezogenen Antisemitismus (‚Durch die israelische Politik werden mir Jüd:innen immer unsympathischer‘) abbilden lässt, können die Triebfaktoren antisemitischer Einstellungsmuster jenseits der konfessionellen Selbstverortung der Befragten beleuchtet werden. Dabei bestätigt sich das auf Grundlage der Leipziger Autoritarismus-Studie (Decker und Brähler 2020) gezeichnete Bild: Sowohl der tradierte als auch der israelbezogene Antisemitismus findet in der Gruppe der Individuen, die sich selbst als Muslim:innen identifizieren, die höchsten Zustimmungswerte (Abb. 11).

Gänzlich überraschend, sind solche empirischen Muster erst einmal nicht. Folgt man der Perspektive der *Social Identity Theory* (Tajfel 1982), dann basieren gruppenbezogene Ressentiments immer auch auf der Konstruktion kollektiver Identitäten und einem rigiden Kategorisierungsprozess, die dazu führen können, dass die ‚religiösen Anderen‘ negativen Attributen belegt werden. Diese kognitive Operation wird in Gang gesetzt, wenn Individuen sich und die ‚Anderen‘ als Mitglieder sich gegenseitig ausschließender Sozialgruppen begreifen (Tajfel und Turner 1979, S. 39–41). Bezüge auf die eigene Religion sind dabei als wirkmächtige Quellen kollektiver Identitätsformationen anzusehen und werden bis in die Gegenwart zur Etablierung exklusiver Gruppenidentitäten genutzt (Welzel und Inglehart 2019). Die Abwertung der Anderen und die Identifikation mit der angeblich erhabenen Eigengruppe ist zudem eine bequeme Strategie zur Erreichung einer positiven Distinktion und entspringt dem menschlichen Bedürfnis nach einer positiven sozialen Identität (Geschke 2012, S. 36–37; Tajfel und Turner 1979, S. 40). Die Identifikation mit einer Religionsgemeinschaft liefert allerdings für sich genommen wenig Rückschlüsse darüber, welche Bedeutung die Religion im Leben der Befragten einnimmt. Ein Faktor den es somit zu berücksichtigen gilt, ist die *Religiosität der Individuen*.

Ob und inwiefern die Religiosität eines Individuums als Quelle gruppenbezogener Ressentiments fungieren kann, ist allerdings stark umstritten. Bereits Allport (1971, S. 444) betonte, dass dem Verhältnis von Religion und Vorurteilen eine paradoxe Beziehung innewohnt. Auf der einen Seite habe Religion das Potenzial, Vorurteilen entgegenzuwirken, weil sich in allen Weltreligionen Anrufungen zur Solidarität und Geschwisterlichkeit finden.

Auf der anderen Seite könne aber auch nicht verschwiegen werden, dass Religionen historisch betrachtet eine Legitimationsgrundlage für Gräueltaten, Verfolgung und Brutalität gegenüber Andersgläubigen und -denkenden lieferten und bis heute liefern. Das Verhältnis von Religion und gruppenbezogener Ressentiments, lässt sich nur sinnvoll untersuchen, wenn die *Auslegung der eigenen Religion* und die Rolle, die sie im persönlichen Leben spielt, genauer beschreiben wird (Allport 1971). Starke Ressentiments sind vor allem unter denjenigen Gläubigen zu erwarten, die zu einer *dogmatisch-fundamentalistischen Auslegung* ihrer Religion tendieren (Altemeyer und Hunsberger 2004). Dies würde sich dann mit der *Selective Intolerance Hypothesis* (Rowatt et al. 2009) decken. Religiös motivierte Ressentiments sind immer dann wahrscheinlich, wenn diskriminierende Handlungen gegen spezifische Gruppen von religiösen Doktrinen und Autoritäten legitimiert werden. Und so überrascht es wenig, dass mehrere Studien belegen können, dass *fundamentalistische Submilieus*, die sich im Besitz der absoluten Wahrheit wähnen und an eine buchstabengetreue Richtigkeit ihrer heiligen Schriften glauben, zu akzentuierten Ressentiments gegenüber den ‚religiösen Anderen‘ tendieren (u. a. Doebler 2014; Pickel et al. 2020; Yendell und Huber 2020). Ob in diesem Zusammenhang wirklich die (fundamentalistische) Religiosität von Individuen, die entscheidende Variable ist, ist umstritten. Bereits Allport (1971) äußerte die Vermutung, dass die Zugehörigkeit zu Religionsgemeinschaften für Menschen mit autoritären Charakterstrukturen (Adorno 1976) attraktiv sein kann, da sie so ihre Ich-Schwäche hinter einer mächtig wirkenden Wir-Gruppe verstecken können. Mit dem Begriff des autoritären Charakters beschrieb Adorno (1976, S. 45–46) eine individuelle Disposition zur unkritischen Haltung gegenüber der Eigengruppe und Abwertung von Fremdgruppen. Dieses Syndrom setzt sich aus verschiedenen Charakterzügen zusammen und umfasst u. a. den Konventionalismus, die autoritäre Unterwürfigkeit sowie die autoritäre Aggression. Das autoritäre Syndrom ist für Adorno (1976, S. 11–12, 173) die zentrale Quelle für minoritätsfeindliche bzw. antisemitischer Ressentiments. Eine Wirkungszusammenhangsvermutung, die bis heute immer wieder bestätigt werden konnte (siehe Decker et al. 2018).

Wie bereits erwähnt, sind viele Muslim:innen in Deutschland immer wieder Stigmatisierungen und Diskriminierungen ausgesetzt (Wetzel 2014, S. 3, 10, 21). Es kann nicht ausgeschlossen werden, dass solche verletzenden Erfahrungen selbst einen Ausgangspunkt für die Aufwertung der Eigen- und Abwertung von Fremdgruppen liefern (Mansel und Spaiser 2010, S. 4). Ein weiteren Faktor den es zu berücksichtigen gilt, ist somit die eigene *Diskriminierungserfahrung*. Im ungünstigsten Falle kommt es zu einer ‚Betroffenenkonkurrenz‘, was dann passieren kann, wenn Muslim:innen zur Einschätzung gelangen, dass die deutsche Mehrheitsgesellschaft mit dem Antisemitismus deutlich sensibler umgeht, als mit den Feindseligkeiten, die ihnen entgegenschlagen (Unabhängiger Expertenkreis Antisemitismus 2017, S. 78, 191–192). Wetzel (2014, S. 21) argumentiert in diesem Zusammenhang, dass Diskriminierung, Rassismus, und Ausgrenzungserfahrungen dem radikalen Islamismus das Spiel erleichtert.

Interreligiöser Dialog und Begegnungen mit Jüd:innen könnten dem entgegenwirken, solange antisemitische Stereotype noch den Status unreflektierter Ideologiefragmente besitzen (Unabhängiger Expertenkreis Antisemitismus 2017, S. 192). Diese Einschätzung steht im Einklang mit der Kontakthypothese aus der Sozialpsychologie. In dem Klassiker *Die Natur des Vorurteils* argumentierte Allport (1971), dass regelmäßiger Intergruppenkontakt unter günstigen Voraussetzungen dem Abbau von Ressentiments zuträglich sein kann. Wahrscheinlich sei ein vorurteilsreduzierender Effekt vor allem dann, wenn zwischen den beteiligten Personen ein egalitärer Status herrscht und gemeinsame Ziele vorliegen, deren Erreichung durch kooperatives Verhalten verfolgt werden. Vorteilhaft ist es zudem, wenn die politischen Institutionen und Autoritäten einer Gesellschaft diesen Kontakt unterstützen (Allport 1971, S. 285–286). Eine Meta-Analyse von Pettigrew und Tropp (2006) konnte die Annahmen der Kontakthypothese plausibilisieren – wobei zu betonen ist, dass Intergruppenkontakten vor allem dann ihre Wirkung entfachen, wenn sie als positiv erlebt werden.

In einem Regressionsmodell haben wir diese alternativen Erklärungsfaktoren berücksichtigt und kontrollieren zu dem eine ganze Reihe von Kontrollvariablen wie die Selbstverortung auf der Links-rechts-Skala, den Wohnort, das Herkunftsland, das Bildungsniveau sowie das biologische Geschlecht und das Alter der Befragten. Die abhängige Variable dieser Analyse ist eine Antisemitismus-Skala, die wir aus der Zustimmung zum tradierten und israelbezogenen Antisemitismus zusammengesetzt haben.

**Figure 12:**
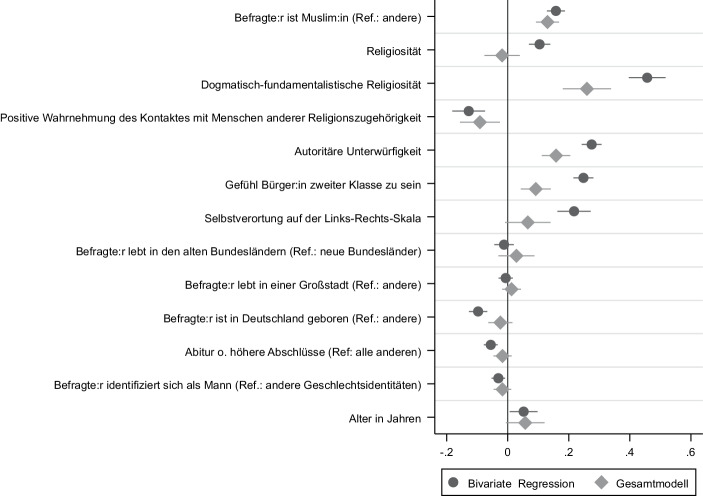

Welche Faktoren begünstigen nun aber antisemitische Einstellungsmuster? Und vor allem: Hat die Selbstidentifikation als Muslim:in überhaupt einen Effekt auf antisemitische Einstellungsmuster, wenn auf die zuvor theoretisierten Einflussfaktoren in der empirischen Analyse mitberücksichtigt werden? Wie den Ergebnissen der OLS-Regression in Abb. 12 entnommen werden kann, ist basale Religiosität – gemessen an der Selbstdeklaration als religiöse Person – kein entscheidenden Triebfaktor antisemitischer Einstellungen (β = −0,018, *p* = 0,534). Religiosität per se scheint gegenwärtig keine Quelle des Antisemitismus zu sein. Allerdings kann der individuellen Religiosität auch keine immunisierende Wirkung zugesprochen werden. Entscheidend ist die Auslegung der eigenen Religion. So erweisen sich im Regressionsmodell dogmatisch-fundamentalistische Auslegungen der eigenen Religion als der wirkungsmächtigste Triebfaktor antisemitischer Ressentiments (β = 0,259, *p* = 0,0001). Ungebrochen ist bis heute zudem der Nexus zwischen dem Autoritarismus und antisemitischen Einstellungsmustern (β = 0,158, *p* = 0,001). Ferner kann plausibilisiert werden, dass das Gefühl ein:e Bürger:in zweiter Klasse zu sein in Teilen der Bevölkerung eine antisemitische Reaktanz hervorruft (β = 0,091, *p* = 0,0001), während eine positive Wahrnehmung des Kontaktes mit den ‚religiösen Anderen‘ antisemitischen Einstellungen entgegenwirkt (β = −0,091, *p* = 0,006). *Hervorzuheben ist jedoch auch, dass selbst unter Berücksichtigung dieser Faktoren, der Nexus zwischen einer Selbstidentifikation als Muslim:in und antisemitischen Einstellungsmustern (β* *=* *0,130, p* *=* *0,00019) bestehen bleibt.*

**Figure 13:**
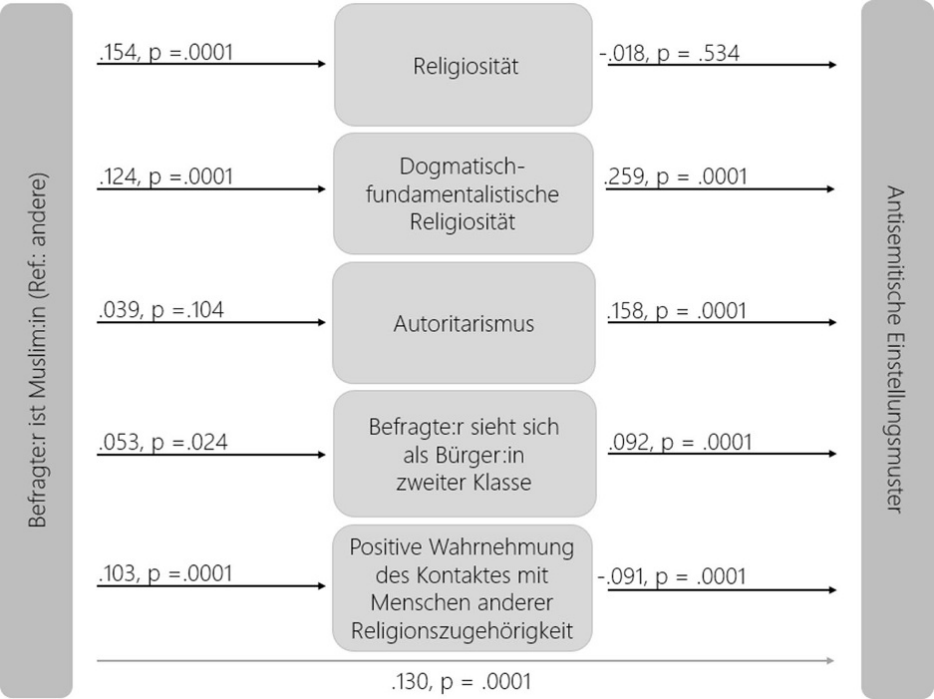

Nun können die dargelegten Drittfaktoren auch als vermittelnde Faktoren zur Erklärung, warum eine höhere Prävalenz antisemitischer Einstellungen unter Muslim:innen vorliegt, genutzt werden. Diese Möglichkeit prüfen wir mit einer Mediationsanalyse. Dafür behandeln wir die alternativen unabhängigen Variablen in einem zweiten Schritt als Mechanismen, die erklären könnten, warum eine akzentuierte Empfänglichkeit für antisemitische Einstellungsmuster unter Muslim:innen beobachtet werden kann (Hayes 2017). Wie der Abb. 13 entnommen werden kann, gelangen Muslim:innen häufiger zu der Einschätzung, dass sie in Deutschland als Bürger:innen zweiter Klasse behandelt werden (β = 0,053, *p* = 0,024). Sie sind religiöser als der Durchschnitt der Gesellschaft (β = 0,154, *p* = 0,0001) und sind zudem empfänglicher für dogmatisch-fundamentalistische Auslegungen der eigenen Religion (β = 0,124, *p* = 0,0001). Hierbei handelt es sich jedoch um eine Minderheit unter den Muslim:innen und so kann beobachtet werden, dass die Mehrheit der Muslim:innen die Begegnungen mit den ‚religiösen Anderen‘ als etwas positives erlebt (β = 0,103, *p* = 0,0001). Eine größere Neigung zu autoritären Charakterstrukturen können hingegen ausgeschlossen werden (β = 0,039, *p* = 0,104). Muslim:innen unterscheiden sich in dieser Frage nicht vom Mainstream der Gesellschaft.

Einer dieser vier potenzialen ‚Pfade‘ stellt sich hierbei also als irrelevant heraus: *Die im Vergleich zu anderen Konfessionen stärkere Religiosität von Muslim:innen ist nicht der Grund für die stärkere Befürwortung antisemitischer Aussagen *(β = −0,018, *p* = 0,534). Letztlich bestehen zwei vermittelnde Faktoren, die zu einer höheren Prävalenz antisemitischer Einstellungsmuster beitragen und ein vermittelnder Faktor, der zu einer Nivellierung in den Einstellungsunterschieden zwischen Muslim:innen und Nicht-Muslim:innen beiträgt.

Die *stärkere Bejahung dogmatisch-fundamentalistischer Auslegungen der eigenen Religion *(β = 0,259, *p* = 0,0001) und die *Perzeption in Deutschland als Bürger:in zweiter Klasse zu behandelt zu werden* (β = 0,092, *p* = 0,0001)^11^, sind die zentralen Triebfaktoren, auf die sich das höhere Niveau antisemitistischer Einstellungen unter Muslim:innen zurückführen lässt. Interreligiöse Begegnungen haben hingegen das Potenzial diesem Trend entgegenzuwirken (β = −0,091, *p* = 0,0001). Muslim:innen, die Begegnungen mit Mitgliedern anderer Religionen als etwas positives erleben, sind weniger empfänglich für antisemitische Einstellungsmuster.^12^

Der stärkste Mediationseffekt geht von einer dogmatisch-fundamentalistischen Auslegung der eigenen Religion aus. Erwähnenswert ist ferner, dass eine partielle Mediation vorliegt. Es besteht jenseits der skizzierten Mediationseffekte ein direkter Effekt von der Selbstidentifikation als Muslim:in auf antisemitische Einstellungsmuster (β = 0,130, *p* = 0,0001). Dies spricht dafür, dass der tradierte und israelbezogene Antisemitismus in einem Submilieu deutscher Muslim:innen zu einem integralen Bestandteil der eigenen Gruppen-Identität avanciert ist (Dantschke 2010). Die empirischen Ergebnisse legen somit nahe, dass der Faktor ‚Religion‘ für die Formation antisemitischer Einstellungsmuster nicht unterschätzt werden sollte. Hierbei ist es nicht Religiosität an sich, wie die Selbstdeklaration als religiöse Person, sondern eine grenzziehende religiös-kollektive Wir-Identität (siehe auch Hößl 2019) und vor allem *dogmatisch-fundamentalistische Auslegungen der eigenen Religion*, die unter Muslim:innen – aber auch in anderen Religionsgemeinschaften – eine Feindschaft gegenüber Jüd:innen begünstigt (Pickel et al. 2020). Letztlich wäre diese ohne die religiöse Zugehörigkeit als Identitätsbezug gar nicht verfügbar.^13^

## Fazit – Notwendige Differenzierungen: Muslimischer Antisemitismus als eine Facette des Antisemitismus in Deutschland

Die Ergebnisse geben Hinweise auf notwendige Differenzierungen mit Blick auf die Verbreitung antisemitischer Ressentiments. *Antisemitismus ist in Deutschland – wie auch in vielen europäischen Nachbarstaaten – längst kein Relikt der Vergangenheit. *Ausgehend von einer nachweisbaren starken Betroffenheit von Jüd:innen in Deutschland und Europa darf die auf den ersten Blick relativ niedrige Verbreitung antisemitischer Ressentiments in Bevölkerungsumfragen nicht als Beruhigung fehlgedeutet werden. So gibt es kaum mehr Menschen jüdischen Glaubens in Deutschland, die nicht Erfahrungen der Diskriminierung und Abwertung kennen. Die Situation in Deutschland ist von einem „Antisemitismus ohne Antisemiten“ (Marin 1979) geprägt. Zu dieser Einschätzung gelangt man allerdings nur, wenn die latente Zustimmung und unterschiedlichen Formen des Antisemitismus nicht berücksichtigt werden. Tut man dies, so ist zu resümieren, dass Antisemitismus verbreiteter ist als angenommen wird. Zwar gibt es gesamtgesellschaftlich keine Mehrheit, die für Antisemitismus empfänglich ist, von einer kleinen Minderheit kann jedoch auch nicht die Rede sein. Blickt man auf die hohe Betroffenheit der in Deutschland (und anderorts) lebenden Jüd:innen, dann ist Antisemitismus als gesamtgesellschaftliches Problem zu realisieren.

Wie schon angedeutet, hat der Antisemitismus in Deutschland *unterschiedliche Formen* angenommen. Neben dem tradierten Antisemitismus sind insbesondere der sekundäre bzw. schuldabwehrende Antisemitismus sowie der israelbezogene Antisemitismus weitverbreitet. Die Differenzierung zwischen verschiedenen ‚Antisemitismen‘ ist auch hinsichtlich seiner Trägergruppen bedeutsam. Unsere Analysen zeigen, dass der schuldabwehrende Antisemitismus eine Domäne ideologisch-rechtsgerichteter Menschen ist, während antisemitischen Aussagen mit einem starken Israelbezug vor allem unter Muslim:innen verbreitet sind. Besonders an dieser Stelle lassen sich unter Muslim:innen in Deutschland höhere Zustimmungswerte als unter Mitgliedern anderer Religionsgemeinschaften oder Konfessionslosen verzeichnen. Allerdings fällt auch der tradierte Antisemitismus unter Muslim:innen stärker aus als im Durchschnitt der Bevölkerungsdurchschnitt – wenn auch auf einem deutlich niedrigeren Niveau.

Gleichwohl ist vor allzu pauschale Aussagen über Muslim:innen zu warnen, unterliegt man sonst doch der Gefahr einer rassistisch angehauchten Zuschreibung (auch Arnold 2019). Unsere Analysen zeigen, dass ein differenzierter Blick von Nöten ist: Während beispielsweise Mitglieder sunnitischer Glaubensgemeinschaften stärker für antisemitische Aussagen empfänglicher sind, fallen antisemitische Ressentiments unter Alevit:innen deutlich weniger akzentuiert aus. Zudem können die Hintergründe einer stärkeren Empfänglichkeit für antisemitische Aussagen variieren. Beispielsweise kann Antisemitismus unter Muslim:innen genauso herkunfts- (z. B. Antizionismus) wie religionsgebundenen (Antijudaismus) Ursprungs sein. Auf letzteres deutet der antisemitismusfördernde Effekt einer dogmatisch-fundamentalistischen Auslegung der eigenen Religion. Solche rigiden Auslegungen der eigenen Religion existieren auch in anderen Religionsgemeinschaften, allerdings fällt der Anteil von dogmatisch-gläubigen Muslim:innen höher aus als unter den Angehörigen christlicher Konfessionen.

Es bedarf ferner genauerer Analysen, inwieweit eigene Diskriminierungserfahrungen die Formation antimuslimischer Einstellungsmuster unter Muslim:innen begünstigen. Unsere Analysen deuten darauf hin, dass viele Muslim:innen zu der Einschätzung gelangen, dass sie in Deutschland wie Bürger:innen zweiter Klasse behandelt werden. Diese Diskriminierungserfahrungen- oder Wahrnehmungen scheinen antisemitische Affekte nach sich ziehen zu können. Hier finden sich möglicherweise intersektionale Abwertungserfahrungen, die auch Jüd:innen in Deutschland machen. Das Problem: Aus der gemeinsamen Ablehnung durch Teile der Bevölkerung entsteht keine gemeinsame Interessenkoalition zwischen Muslim:innen und Jüd:innen. Religiöse wie historisch-regionale Gründe stehen einer solchen Allianzbildung entgegen. Statt einer solidarischen postmigrantischer Gesellschaft scheint viel eher eine ‚*Betroffenenkonkurrenz‘* zu überwiegen.

Trotz der stärkeren Verbreitung antisemitischer Ressentiments unter Muslim:innen besteht kein Stillstand in der beschriebenen Gemengelage. Es ist weder so, dass das Überleben dogmatischer Religiosität angesichts fortschreitender Säkularisierungstrend gesichert ist, noch dass es sich bei antisemitischen Ressentiments um kulturelle Erscheinungsformen handelt, die gegenüber einem Wandel immun sind (Shooman 2014). Der Vergleich zu den Herkunftsländern zeigt Anpassungsprozesse an die Gesellschaft in der man lebt – und damit auch ein *Abbau von „erlerntem“ Antisemitismus*. Hier werden auch Interventionsmöglichkeiten erkennbar, welche in Wissensvermittlung, Kontaktherstellung und jüdisch-muslimischen Dialog liegen. Gleichzeitig gibt es auch andere – unerfreulichere – Anpassungsprozesse: Es zeigen sich *Tendenzen einer gewissen Übernahme des schuldabwehrenden Antisemitismus*.

Gesamtgesellschaftlich bleiben der Autoritarismus und die daraus resultierende Offenheit für Verschwörungsnarrative die dominanten Erklärungsfaktoren für den Antisemitismus. Hinzu kommt die Wirkmacht rechter Ideologien. Am Ende des Tages steht der Antisemitismus unter Muslim:innen dann weniger im Kontrast, denn im Einklang mit dem Antisemitismus der Mehrheitsgesellschaft.

Zentrales Ergebnis unserer Analysen bleibt: Es existieren unterschiedliche Erklärungsstrukturen und unterschiedliche Trägergruppen verschiedener Formen des Antisemitismus. Der Formenwandel und die Formenerweiterung des Antisemitismus belegt seine historische Überlebenskraft. Auch heute noch ist er existent und sorgt dafür das der Großteil der in Deutschland lebenden Jüd:innen antisemitische Diskriminierungserfahrungen erleiden muss. Dabei finden sich in den Betroffenenbefragungen unterschiedliche Referenzgruppen von denen solche Diskriminierungen ausgehen. Zusammen mit den gezeigten unterschiedlichen Ressentimentstrukturen in Bevölkerungsumfragen wird deutlich, dass eine *differenzierte, die aufgezeigten Unterschiede aufnehmende, empirische Analyse des heutigen Antisemitismus* unumgänglich ist, will man ihm erfolgreich entgegentreten. Es sind mehr Kenntnisse über unterschiedliche Formen des Antisemitismus notwendig, um auf Basis des Ermittelten geeignete Instrumente für seine Bekämpfung und Prävention entwickeln zu können (auch Arnold 2019, 2020). Man muss dabei aufpassen, Muslim:innen nicht vorschnell und pauschal zum Opfer von globalen Zuschreibungen als muslimische Antisemit:innen werden zu lassen. Man darf aber ebenfalls nicht in die Falle der (bewussten) Ignoranz eines ‚islamisierten‘ Antisemitismus laufen. Schließlich gilt es jede Form von Antisemitismus konsequent und zielgenau zu bekämpfen.

## Footnotes

1 Wir verwenden im vorliegenden Text die geschlechtsinklusive Form Jüd:innen.

2 Der Artikel entstand im Rahmen des von Prof. Dr. Susanne Pickel geleiteten BMBF-Projektverbundes „Radikaler Islam versus Radikaler Anti-Islam“ sowie des BMBF geförderten Forschungsprojektes „Politischer Kulturwandel? Legitimität der Demokratie und gesellschaftlicher Zusammenhalt in Zeiten verstärkten Populismus und steigender Islamablehnung“ im Forschungsinstitut Gesellschaftlicher Zusammenhalt, Projektkennung: LEI_F_08. Zudem werden Daten des aus Mittel der DFG und des SNF geförderten Deutsch-Schweizer Projektes „*Konfigurationen individueller und kollektiver religiöser Identitäten und ihre zivilgesellschaftlichen Potenziale*“ (KONID) verwendet. Wir danken für die Förderungen von BMBF und DFG.

3 Exemplarisch zeigte sich dies an einer prominent besetzten Podiumsdiskussion im Jüdischen Museum zum gleichnamigen Titel. Siehe: https://www.jmberlin.de/diskussion-importierter-antisemitismus.

4 Die Kategorie ‚muslimisch‘ ist in der vorliegenden Nutzung keine rassistische Fremdzuschreibung. Quantitative-empirische Aussagen sind nur über Kategorienbildungen möglich. Letztere erlauben einen Breitenüberblick, der von Einzelfällen abstrahiert und zu einer Verallgemeinerung der Aussagen führt. Die Ergebnisse deuten auf Probleme in der Gesamtbevölkerung und ihrer politischen Kultur, ohne dabei Fremdzuschreibungen vorzunehmen. Als Fremdzuschreibungen können allein geäußerte Vorurteile gegenüber Muslim:innen interpretiert werden (hierzu siehe Öztürk und Pickel 2019).

5 Eine Schlüsselrolle spielte ein Radiosender aus der brandenburgischen Kleinstadt Zeesen. Zwischen 1939 und 1945 ging der Sender allabendlich auf Arabisch, Persisch und Türkisch auf Sendung um den Antisemitismus nationalsozialistischer Prägung in der islamischen Welt salonfähig zu machen. Tatsächlich gelang es dem beliebten Sender die Diktion antisemitischer Weltverschwörungsmythen sowie eine auf Vernichtung ausgelegte Rhetorik gegenüber dem damals im Entstehen begriffenen Staat Israel zu popularisieren – was den Nazis auch deshalb glückte, weil sie dem Antisemitismus durch heavy-rotation der antijüdischen Passagen des Korans eine theologische Patina verliehen (Küntzel 2019).

6 Für eine Dokumentation der antisemitischen Karikaturen der Zeitung sei auf eine Recherche der Kreuzberger Initiative gegen Antisemitismus verwiesen: https://www.kiga-Berlin.org/uploads/Material/Kiga%20Sonderbeilage%20Vakit.pdf.

7 Die Anti-Defamation League ist eine amerikanische Menschenrechtsorganisation, die sich global gegen Antisemitismus – also gegen die Diskriminierung und Diffamierung von Jüd:innen – einsetzt. Die Themenschwerpunkt der Anti-Defamation League sind vielfältig, seit ihrer Gründung im Jahr 1913 hat die Organisation immer wieder Bevölkerungsumfragen in den USA finanziert. Die Surveys zielen darauf ab das Ausmaß der Befürwortung antisemitischer Stereotype in der Bevölkerung aufzuspüren. Seit den 2010er Jahren hat die Anti-Defamation League begonnen diese Umfragen zu internationalisieren. Zwischen Juli 2013 und Februar 2014 wurden die Bevölkerungen von insgesamt 102 Nationen *face-to-face* oder telefonisch repräsentativ befragt. 2015 und 2019 wurde die Befragung in mehreren Ländern wiederholt. Wir nutzen die Daten, die zwischen 2013 und 2014 erhoben wurden, um das größte mögliche Sample untersuchen zu können (Anti-Defamation League 2019). Methodische Details können der Internetseite der Anti-Defamation League (2019) entnommen werden. Für eine Kritik des methodischen Vorgehens der Studie sei auf Ranan (2018) verwiesen.

8 Diese Interpretation wird gelegentlich kritisch hinterfragt. Gleichzeitig erscheint das Argument einer Nichtablehnung als latente Zustimmung vor dem Hintergrund der Schärfe der eingesetzten Statements und angesichts einer jahrelangen politischen Bildung gegen Antisemitismus doch plausibel und wir folgen dieser Verwendung.

9 Die beträchtlichen Unterschiede zwischen Sunnit:innen und Schiit:innen haben vermutlich weniger mit Glaubensunterschieden als mit den Spezifitäten ihrer Migrationsgeschichte zu tun. Die große Mehrheit der in Deutschland lebenden Schiit:innen stammen aus der Iran und sind als Geschäftsmänner bzw. Geschäftsfrauen, zu Studienzwecken und – nach der islamischen Revolution 1979 – aus politischen Gründen nach Deutschland eingewandert. Nicht selten stammen sie aus der Mittel- und Oberschicht des Irans und/oder sind selbst vor einem islamistischen Regime geflohen (Farrokhzad 2010).

10 Dass Alevit:innen selbst zu einer starken Bedrohungsperzeption gegenüber dem (sunnitischen) Islam tendieren, ist ihren gesellschaftlichen Diskriminierungserfahrungen als heterodoxe Religionsgemeinschaft in der Türkei geschuldet sein. Sowohl im osmanischen Reich als auch in der Geschichte und Gegenwart der türkischen Republik waren und sind die Alevit:innen – neben Alltagsdiskriminierungen – immer wieder Gewaltexzessen und islamistisch motivierten Pogromen (z. B. das Massaker in Sivas 1994) ausgesetzt (statt vieler Karakaya-Stump 2017).

11 An dieser Stelle weichen unsere Befunde von der Einschätzung Jikelis ab. Jikeli (2019, S. 56) argumentiert, dass das im Vergleich zu den Herkunftsgesellschaften geringere Niveau antisemitischer Einstellungen unter Muslim:innen in europäischen Einwanderungsgesellschaften, die These ad absurdum führt, dass Antisemitismus erst durch Diskriminierungserfahrungen in Deutschland entstehe. Auch wir glauben nicht, dass ein direkter kausaler Nexus zwischen Diskriminierungserfahrungen und der Formation antisemitischer Einstellungen gegeben ist – zumal die Frage im Raum steht, warum ausgerechnet Jüd:innen als Sündenbock für die eigenen Diskriminierungserfahrungen verantwortlich gemacht werden. Gleichwohl können Ausgrenzungs- und Diskriminierungserfahrungen die Identifikation mit der Eigengruppe erhöhen und zu einer erhöhten Attraktivität von islamistischen Gruppierungen führen, was – unter ungünstigen Bedingungen – einer (antisemitischen) Radikalisierung in die Hände spielen kann (Ebner 2017; Schneider et al. 2020).

12 Diese auf den ersten Blick widersprüchlichen Mediationseffekte lassen sich auflösen, wenn in der Gruppe der Muslim:innen eine Binnendifferenzierung vorgenommen wird. Korrelationen zeigen, dass Muslim:innen, die zu einer dogmatisch-fundamentalistischen Auslegung ihrer Religion tendieren, ihre Begegnungen mit Mitgliedern anderer Religionen seltener positiv erleben (r = −0,148, p = 0,004) und häufiger zur Einschätzung gelangen, dass sie in Deutschland als Bürger:innen zweiter Klasse behandelt werden (r = 0,301, p = 0,0001).

13 Zudem ermöglicht die Differenzierung in Religiosität und im Religionsverständnis Aussagen auch über die Wirkung von Religion, die reine Identitätsmuster übersteigen.
